# Impact of Supply Chain Disruptions and Drug Shortages on Drug Utilization: A Scoping Review

**DOI:** 10.1002/pds.70178

**Published:** 2025-07-02

**Authors:** Araniy Santhireswaran, Shanzeh Chaudhry, Martin Ho, Kaitlin Fuller, Etienne Gaudette, Lisa Burry, Mina Tadrous

**Affiliations:** ^1^ Leslie dan Faculty of Pharmacy University of Toronto Toronto Ontario Canada; ^2^ Angus L. Macdonald Library St. Francis Xavier University Antigonish Nova Scotia Canada; ^3^ Institute of Health Policy, Management and Evaluation University of Toronto Toronto Ontario Canada; ^4^ Mount Sinai Hospital Sinai Health Toronto Ontario Canada

**Keywords:** drug access, drug shortages, drug utilization, scoping review

## Abstract

**Purpose:**

Drug shortages are a growing challenge in health systems across the world. A better understanding of the impacts of shortages on patient drug access and use will guide policies aimed at mitigating shortages. This scoping review aims to summarize observational literature assessing the impact of drug shortages on drug utilization trends.

**Methods:**

We searched Ovid MEDLINE and Ovid EMBASE for studies published between 1946 and September 17, 2024. An extensive grey literature search was conducted through targeted website searches, grey literature databases, and the Google search engine. Observational studies examining the impacts of drug shortages on drug use were included. Study screening and extraction were conducted by two independent reviewers.

**Results:**

We identified 55 published articles and five gray literature reports. Most studies were conducted in North America (*n* = 42, 70%). Population‐level data were most used (*n* = 34, 57%), and most studies used drug prescription data to assess changes in use (*n* = 30, 55%). Most studies reported changes in drug use as a percent change, and the magnitude in decreases ranged from 1% to 99%. Many different data sources, methods, and measures were used to study the impact of drug shortages on drug utilization, and a broad range of decreases in drug utilization following the shortages were reported.

**Conclusions:**

It is important to synthesize findings across studies to understand how different drugs and settings are affected by shortages. The findings here will inform future studies aimed at filling this knowledge gap, ultimately yielding real‐world evidence that can guide policy decisions to address drug supply challenges.

**Summary**

- This scoping review synthesizes findings from 55 published articles and 5 grey literature reports, revealing that drug shortages lead to a broad range of decreases in drug use, with reported reductions varying from 1% to 99%.
- The study also highlights the diverse methodologies, data sources, and metrics used to assess utilization changes, which can complicate comparability across studies. Prescription data were the most commonly used source, alongside other sources including administration data, claims data, and wholesale purchases.
- Importantly, it identifies key drug classes and shortage events that have been extensively studied, as well as gaps in settings and study designs. Angiotensin receptor blockers were the most studied drug class, with valsartan being the most studied drug.
- By summarizing current evidence on the impact of drug shortages on utilization, this study can guide future research toward standardizing methods for evaluating utilization trends during shortages.
- These insights may support the development of more resilient drug supply chains, thereby improving patient care during shortages.

## 1 Introduction

Drug shortages have become a complex global issue, impairing patient care and burdening the healthcare system. The number of drugs facing shortages in two or more countries has increased by 101% between September 2021 and January 2024 [1]. A drug shortage is a situation where supplies of essential medicines and health products do not meet patient needs as defined by the World Health Organization (WHO) [2]. Drug shortages cause substantial disruptions in patient treatment and can lead to severe consequences, including delayed treatment, adverse drug events, medication errors, increased costs, hospitalization, and even mortality [3, 4, 5]. In addition, drug shortages further strain the healthcare system through increased labour, recovery and procurement costs, and prolonged patient recovery times, all tied to finding appropriate treatment alternatives [6].

Despite the many negative implications of drug shortages, there is limited literature on the impact of drug shortages on population‐level drug use and access. Using real‐world data to analyze population‐level drug use trends can offer valuable insights into the underlying mechanisms of the drug supply chain and potential shortages [7]. A recent scoping review aimed at thematically analyzing all literature related to drug shortages found that, out of 430 articles, only 50 were retrospective or observational studies [8]. Although these 50 studies contribute valuable insights, it is still unclear how many studies specifically examine the impacts of shortages on patient drug use and access to medicines. It is important to highlight this knowledge gap and determine the quantity of studies focusing on specific effects of shortages on drug access, usage patterns, and supply stability and how they have been conducted. Summarizing this information will help guide more high‐quality studies to deepen our understanding of these critical aspects.

To shed light on this knowledge gap, we conducted a scoping review of observational studies analyzing drug utilization trends during supply chain disruptions and demand‐related shortages. We aimed to answer the question, “How has the impact of pharmaceutical product shortages on utilization trends been studied in the literature?” This scoping review (1) identified drugs that have been studied during shortages and described their characteristics, (2) examined the jurisdictions and healthcare settings where these studies were conducted, and (3) assessed how changes in drug use and the extent of their impact were reported. This scoping review differs from existing reviews by specifically quantifying the impact of shortages on population‐level drug use and examining how observational studies in this area are designed and conducted. The scoping review methodology was well‐suited to achieving these objectives, as it allowed for the exploration of a broad spectrum of literature to understand how studies evaluated and reported on drug use during shortages [9]. Additionally, this approach helped to identify the breadth of available evidence on the topic, as well as key gaps in the research [10].

## 2 Methods

This review used the JBI framework for scoping reviews, which is grounded in the six‐stage process originally proposed by Arksey & O'Malley (2005) and refined by Levac et al. (2012) [11, 12, 13]. This manuscript adheres to the PRISMA‐ScR guidelines to ensure proper reporting standards [14]. This scoping review was registered in OSF [15] and followed an a priori protocol [16]. Detailed methods have been previously published [16].

For this review, both published and unpublished observational studies, including cohort, case–control, and self‐controlled studies, were included. Our review question focuses on the impacts of drug shortages on drug use in large populations. Therefore, case series and case reports will be excluded since they only recount a few occurrences of the outcome instead of analyzing large populations and lack comparators. Moreover, randomized controlled trials (RCTs) will be excluded given that randomization is infeasible for our review question, and we aim to better understand the real‐world impact of shortages. Literature reviews (i.e., systematic, scoping, narrative, etc.), meta‐analyses, study protocols, opinion pieces (i.e., editorials, commentaries, letters, etc.), conference abstracts, and studies with unavailable full‐text articles will be excluded due to the lack of primary research analysis. A search for grey literature was also conducted to include unpublished observational studies and reports from governmental and healthcare organizations. Table 1 specifies the eligibility criteria.

**TABLE 1:** Eligibility criteria.

Component	Description
Participants	Any population using prescription drugs, over‐the‐counter drugs, vaccines, therapy products, pharmaceutical solutions, and sterile solutions, that have encountered a supply chain disruption.
Concepts	Drug supply chain disruptions included any shortages, product discontinuations, drug recalls, market withdrawals, and demand‐related shortages.
Context	Studies examining the impact of drug supply disruptions on drug utilization and access that utilized population‐level drug utilization data from various healthcare settings and regions, including wholesaler purchasing, prescription dispensing, claims, and drug administration data. Studies conducted in any healthcare setting and region. Studies published in any language.
Types of Sources	Published observational studies (i.e., cohort studies, case–control studies, cross‐sectional studies, self‐controlled studies). Unpublished observational studies and reports from relevant healthcare and government organizations.

The search was systematically conducted in Ovid MEDLINE from 1946 to September 17, 2024, and in Ovid EMBASE from 1947 to September 17, 2024. An initial search was conducted on September 20, 2023. The search strategy was updated and rerun in Ovid MEDLINE and Ovid EMBASE on September 17, 2024. No limits were placed on the search. During the search update, additional keywords were included to ensure all relevant articles were captured (Appendix A). A grey literature search was conducted using grey literature databases, targeted websites, and the Google search engine (Appendix B). For the Google search engine, targeted queries were used, and the first five pages of search results were examined by an independent reviewer. While scoping reviews typically require searches across three databases, the specificity of our topic and knowledge that this information could be disseminated outside of journal articles led us to perform a more thorough grey literature search instead of including a third database. Reference lists from selected articles and relevant reviews were also hand‐searched backward by browsing reference lists to ensure no pertinent studies were missed.

Covidence Systematic Review Software (Veritas Health Innovation, Melbourne, Australia) was used for deduplication, screening, and extraction. Three authors (AS, SC, MH) independently screened abstracts and full‐texts, and extracted the data ensuring each article was screened and reviewed twice by different authors. Any disagreements were resolved by a third reviewer (MT). The data extraction form was tested on a sample of three studies before extraction was conducted independently. The extracted variables included general article characteristics, study design, data sources, drug characteristics, shortage characteristics, outcomes, key findings, conclusions, and funding sources.

## 3 Results

**FIGURE 1:**
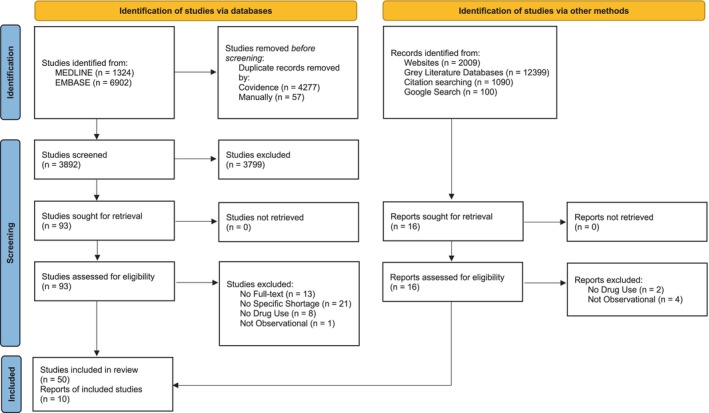
PRISMA flow diagram of study inclusions and exclusions.

Of the 3892 unique articles we identified through the database search, 3799 were excluded in the title and abstract screening. The remaining 93 articles were assessed for eligibility in the full‐text screening, and 43 were excluded, resulting in 50 relevant articles from MEDLINE and EMBASE. The citation hand‐search identified five peer‐reviewed articles which were included in the review. The targeted website search identified 2009 reports, 11 of which were screened for eligibility, and five relevant reports were included. Grey literature databases identified 12 399 reports and no relevant items were found, and the Google search engine search also provided no relevant items. A total of 55 published articles from peer‐reviewed journals and five unpublished reports from grey literature were included in the scoping review. Figure 1 displays the PRISMA flowchart for the database and grey literature search, and Appendix B details the grey literature search documentation. Of the included studies, the main objective of 24 (40%) studies was to assess changes in drug use alone, while 36 (60%) studies had an additional objective. Of these, 23 assessed impacts to clinical outcomes, 11 studied impacts to cost and seven examined changes to clinical practice (Table 2).

**TABLE 2:** Objectives of included studies.

	Objective	*N*	Study ID
Objectives	Utilization only	24 (40)	1, 2, 3, 8, 10, 11, 12, 13, 14, 16, 17, 20, 24, 27, 31, 32, 34, 42, 44, 45, 50, 51, 57, 59
	Utilization and cost	9 (15)	15, 23, 28, 30, 41, 46, 56, 58, 60
	Utilization and clinical outcomes	18 (30)	5, 9, 18, 21, 25, 26, 29, 36, 37, 38, 39, 40, 47, 48, 49, 53, 54, 55
	Utilization and clinical practice	4 (7)	6, 22, 43, 52
	Utilization, cost, and clinical outcomes	2 (3)	7, 35
	Utilization, clinical outcomes, and clinical practice	3 (5)	4, 19, 33

*Note:* See Appendix C for Study ID.

### 3.1 Healthcare Settings

Most studies were conducted in North America (*n* = 42, 70%); seven (12%) were conducted in Europe, six (10%) in Asia, two (3%) in Australia, and three (5%) were global studies. The most common countries studied were the United States (*n* = 35), Canada (*n* = 10), and Japan (*n* = 4). Twenty‐six (43%) studies were conducted in an inpatient healthcare setting, 14 (23%) in outpatient, and 20 (33%) in both inpatient and outpatient. Population‐level data were used by most studies (*n* = 34, 57%); 21 (35%) were single‐center studies, and five (8%) were multicenter studies. Most studies used drug prescription data (*n* = 30); 10 used prescription claims data, 16 used drug administration data, and eight used wholesale purchasing data. IQVIA was the most common database used, including IQVIA's Multinational Integrated Data Analysis (MIDAS), disease analyzer, medical claims data, national sales perspective, total patient tracker, longitudinal patient database, medical research data, and geographic prescription monitor. Twenty‐two studies used chart reviews of healthcare centers to collect data. Table 3 specifies the healthcare setting characteristics of all studies included.

**TABLE 3:** Healthcare setting characteristics.

	Characteristic	*N* (%)	Study ID
Healthcare setting	Inpatient	26 (43)	4, 5, 7, 14, 18, 19, 21, 21, 26, 27, 28, 29, 33, 35, 36, 38, 40, 41, 42, 43, 46, 47, 48, 49, 54, 55
	Outpatient	14 (23)	1, 2, 6, 9, 17, 23, 24, 31, 34, 44, 45, 50, 56, 59
	Both	20 (33)	3, 8, 10, 11, 12, 13, 15, 16, 20, 25, 30, 32, 37, 39, 51, 52, 5357, 58, 60
Healthcare level	Single center	21 (35)	4, 5, 6, 7, 8, 18, 21, 22, 26, 28, 29, 33, 35, 36, 38, 39, 40, 42, 47, 48, 49, 54
	Multicenter	5 (8)	8, 14, 43, 46, 55
	Population	34 (57)	1, 2, 3, 9, 10, 11, 12, 13, 15, 16, 17, 19, 20, 23, 24, 25, 27, 30, 31, 32, 34, 37, 41, 44, 45, 50, 51, 52, 53, 56, 57, 58, 59, 60
Type of data used	Prescription dispensing	28 (47)	2, 3, 8, 15, 16, 17, 21, 22, 24, 25, 27, 28, 31, 34, 35, 37, 39, 40, 41, 42, 43, 44, 45, 46, 48, 50, 53, 56
	Prescription claims	8 (13)	1, 9, 12, 13, 23, 32, 52, 59
	Wholesale drug purchasing	5 (8)	10, 11, 20, 51, 60
	Drug administration	15 (25)	4, 5, 6, 7, 14, 18, 19, 29, 33, 36, 38, 47, 49, 54, 55
	Prescription dispensing & purchasing	1 (2)	58
	Prescription dispensing & administration	1 (2)	26
	Prescription claims & purchasing	2 (3)	30, 57

### 3.2 Shortage Drugs

Most studies examined shortage events (*n* = 42, 70%), 15 (25%) examined recalls, two (3%) assessed market withdrawals, and one (2%) examined shortages and recalls [17] (Table 4). Most events were due to contamination of the drug product (*n* = 17) or an increase in demand (*n* = 12). All studies examined prescription drug shortages, with the exception of one study that assessed the impacts of the ranitidine recall in both prescription and over‐the‐counter wholesale drug purchases [18]. Most articles studied parenteral drug shortages (*n* = 34, 57%), 16 (27%) examined oral drugs, eight (13%) assessed oral and parenteral formulations, and two (3%) assessed oral, parenteral, and other formulations (Table 4). The most common drug class studied was antibiotics (*n* = 14); 10 studied angiotensin receptor blockers, five examined opioids, and six assessed multiple drugs from varying drug classes. Valsartan was the most common drug examined (*n* = 10), and nine of the studies were in response to the July 2018 valsartan recall. Piperacillin/tazobactam was the second most common drug assessed (*n* = 7). Most studies examined a singular shortage event, and six assessed the impacts of multiple shortages.

**TABLE 4:** Shortage and drug characteristics.

	Characteristic	*N* (%)	Study ID
Shortage status	Shortage	42 (70)	1, 4, 5, 6, 7, 9, 12, 14, 15, 18, 19, 21, 22, 23, 26, 27, 28, 29, 30, 31, 33, 34, 35, 36, 38, 40, 41, 42, 43, 44, 45, 48, 49, 51, 52, 54, 55, 56, 57, 58, 59, 60
	Recall	15 (25)	2, 3, 8, 11, 13, 16, 17, 20, 24, 25, 32, 37, 39, 47, 50
	Market withdrawal	2 (3)	46, 53
	Shortages & recall	1 (2)	10
Drug formulation	Oral	16 (27)	3, 9, 11, 13, 15, 16, 17, 20, 24, 25, 31, 32, 34, 37, 39, 50
	Parenteral	34 (57)	4, 5, 6, 7, 8, 12, 14, 19, 21, 22, 26, 27, 28, 29, 30, 33, 35, 36, 38, 40, 41, 42, 43, 44, 45, 46, 47, 49, 51, 52, 53, 54, 55, 57
	Oral & parenteral	8 (13)	1, 2, 18, 23, 48, 58, 59, 60
	Oral, parenteral & other	2 (3)	10, 56

### 3.3 Changes in Drug Use

Most studies reported changes in use of the shortage drug in response to the supply chain event (*n* = 53, 88%), two (3%) reported changes in comparator drugs, and five (8%) reported the number of switches. Most used percent changes to measure changes in drug use (*n* = 46, 77%), three (5%) reported changes in rates of use and 11 reported other changes including number of patients, proportion of shortages with decreases past a set threshold, morphine equivalents, days of therapy (DOT) per 100 days, and monthly defined daily doses/1000 inhabitants/day (DID). Statistical analyses were conducted by 47 studies (78%), while (22%) studies only reported descriptive statistics. Decreases in drug use ranged from 1% to 99% (Appendix C). Of the 39 studies that examined percent decreases of the shortage drug 22 (56%) had decreases less than 25%, two (5%) reported decreases between 26% and 50%, eight (21%) had drops between 51% and 75%, and seven (18%) reported decreases greater than 76%. Table 5 specifies the drug variation metrics used in all studies.

**TABLE 5:** Changes in drug use.

	Characteristic	*N* (%)	Study ID
Measured changes in:	Shortage drug	53 (88)	1, 3, 5, 7, 8, 9, 10, 11, 12, 13, 14, 15, 16, 17, 18, 19, 20, 21, 22, 23, 24, 26, 27, 28, 29, 30, 31, 32, 33, 34, 36, 38, 39, 40, 41, 42, 43, 44, 45, 46, 47, 48, 50, 51, 52, 53, 54, 55, 56, 57, 58, 59, 60
	Comparator drug	2 (3)	4, 49
	Switches	5 (8)	2, 6, 25, 35, 37
Reported changes as:	Percent	46 (77)	1, 4, 6, 7, 8, 9, 11, 12, 13, 14, 15, 16, 17, 18, 20, 21, 23, 24, 25, 26, 28, 29, 31, 32, 33, 34, 35, 37, 38, 40, 41, 42, 44, 45, 47, 48, 49, 50, 51, 52, 53, 54, 55, 57, 58, 60
	Rates	3 (5)	3, 27, 36
	Other	11 (18)	2, 5, 10, 19, 22, 30, 39, 43, 46, 56, 59
Range in percent decreases of shortage drugs^a^	Less than 25%	22 (56)	1, 9, 11, 12, 13, 14, 15, 16, 21, 23, 24, 33, 34, 38, 41, 45, 51, 52, 54, 55, 57, 60
	26%–50%	2 (5)	28, 58
	51%–75%	8 (21)	17, 18, 20, 26, 32, 42, 50, 53
	Greater than 76%	7 (18)	7, 20, 29, 31, 40, 42, 47

^a^ Among studies that reported percent decreases of the shortage drug (*n* = 39).

## 4 Discussion

Our results emphasize that various data sources, analyses, and measures have been used to understand how shortages affect not only drug utilization but also healthcare resource utilization, prescribing practices, clinical outcomes, and costs incurred by the system. An important observation from our review is the variety of methods used to measure changes in drug use. While many studies quantified utilization shifts using percentage changes, others employed rates of use, morphine equivalents, defined daily doses (DID), or the number of affected patients. This heterogeneity makes comparing findings across studies challenging, as different metrics can yield different interpretations of the same shortage. Although we recognize that not all studies can adopt identical measures due to variations in drug characteristics and contexts, establishing some degree of standardization could improve comparability and provide clearer insights into how shortages affect different drugs and healthcare settings. We also noted wide variability in the magnitude of changes in drug use following drug shortages, indicating that shortages manifest in different ways for different drugs and sectors.

Another trend observed was the focus on single‐event shortages in most studies, with only a few assessing the broader impacts of multiple concurrent shortages. Only five studies assessed the impacts of all shortages affecting a region in a set period, which were conducted in the United States, Canada, and the Netherlands (Appendix C). While research on individual events provides valuable insights, there is a notable gap in research examining multiple shortages within a region over time. Addressing this gap could offer a more comprehensive view of how shortages collectively influence healthcare systems. Such an approach would allow for a better understanding of how different shortages manifest across healthcare settings within a region and how shortages in specific drug classes differently affect access and usage patterns. This perspective is essential for grasping the broader, system‐level impacts of recurring shortages and for informing strategies to address these challenges across global healthcare systems. Moreover, high‐profile shortages, such as the 2018 valsartan recall, received considerable research attention which led to nine studies investigating its effect, underscoring the widespread global impact of this particular event. However, other significant shortages may be underreported, particularly for drugs that lack the same level of media coverage or public awareness. Prioritizing research that targets essential drugs for public health—regardless of their profile or media attention—could provide a more balanced understanding of drug shortages and their implications across healthcare.

In terms of healthcare settings, there was a notable underrepresentation of studies conducted in outpatient or community settings, with a majority of research focused on inpatient environments. Drug shortages, however, are not confined to hospitals; they also disrupt care in outpatient settings where medication access is critical to ongoing management of chronic conditions. Broadening research to include outpatient and community settings would provide a more comprehensive picture of the impacts of shortages across all areas of patient care. Our review also revealed a gap in studies that utilized multicenter or population‐level data, with many studies conducted at the single‐center level. While single‐center studies provide valuable insights, their limited scope may restrict generalizability. Multicenter and population‐level studies could yield more representative data, capturing variations across regions and healthcare systems and supporting the development of more comprehensive policy responses to drug shortages.

Our study has both strengths and weaknesses that are important to consider. This scoping review is the first literature review that thoroughly investigates the impacts of drug shortages on drug access and utilization specifically. By contrast, previously published scoping reviews employed broad overarching aims [8, 19]. For example, Tucker et al. [8] aimed to classify all drug shortage literature using a generalized search strategy ((drug AND shortage) OR (medicine AND shortage)), while Phuong et al. [19] focused on shortage impacts on patient outcomes and grouped studies thematically into humanistic, economic, and clinical categories. Both reviews addressed broad questions and relied on thematic categorization. In contrast, our study posed more targeted research questions and used a comprehensive search strategy tailored to identify observational studies examining how shortages affect drug utilization and access. This approach enabled us to thoroughly explore shortage impacts on drug use, characterize drugs and shortage events, and assess the breadth of outcomes influenced by these disruptions. Another strength is that we employed robust JBI methodology in this scoping review and conducted an extensive search of gray literature, including reports from government and health organizations on drug shortages. A limitation of this review is that only two literature databases were searched, whereas it is standard practice for knowledge synthesis projects such as scoping reviews to include three or more databases. This decision was made to ensure we maintained a feasible project while ensuring we could dedicate time to a comprehensive grey literature search instead of adding a third, multidisciplinary database or focused subject database [16].

In conclusion, drug shortages continue to disrupt care delivery, negatively affecting patient clinical outcomes and further burdening the healthcare system. Gaining a better understanding of the extent, frequency, and severity of these shortages is essential for informing policy decisions aimed at alleviating their impact [20]. This scoping review has summarized shortages that have been studied, along with their characteristics, settings, and regions where these studies have taken place, while also highlighting the extent of their impact on drug use and access. Synthesizing this information allows us to compare the effects of different shortages, as they vary in the way they manifest and the consequences they bring. These insights can guide future research on drug shortages and the varying effects of supply disruptions to help shape frameworks and policy decisions related to drug supply issues.

### 4.1 Plain Language Summary

Drug shortages are becoming a bigger problem in health systems worldwide, making it harder for patients to get the medications they need. Understanding how these shortages impact the use of medications can help create better policies to manage and prevent them. In this scoping review, we looked at studies that examined how drug shortages affect medication use. We searched major medical research databases (MEDLINE and EMBASE) for studies published up to September 17, 2024, as well as relevant websites, grey literature databases carrying unpublished works, and Google for additional reports. We included studies that assessed how drug shortages changed how medications were used. We found 55 published studies and 5 reports. Most studies (70%) came from North America. Researchers often used population‐level data and prescription records to measure changes in drug use. In most cases, medication use dropped after a shortage, with decreases ranging anywhere from 1% to 99%. The studies used many different types of data, methods, and measurements to understand these changes. The results showed that drug shortages can lead to a wide range of reductions in medication use. These findings, which show wide variation, highlight the need to summarize information from different studies to better understand how shortages affect patients and health systems. This review can help guide future research and support policymakers in creating strategies to deal with drug shortages and improve access to essential medications.

## Author Contributions

Research design: A.S., K.F., E.G., L.B., M.T.; Screening and data collection: A.S., S.C., M.H.; Data analysis: A.S.; Manuscript writing: A.S., M.T.; Revisions: A.S., S.C., M.H., K.F., E.G., L.B., M.T.

## Conflicts of Interest

The authors declare no conflicts of interest.

## Supporting information


**Appendix S1.** Supporting Information.
