# Supplementary material for: Impact of Supply Chain Disruptions and Drug Shortages on Drug Utilization: A Scoping Review
Source: Pharmacoepidemiol Drug Saf. 2025 Jul 2;34(7):e70178. doi: 10.1002/pds.70178 (PMC12215599; doi:10.1002/pds.70178)
Supplement: Supplementary file 1 — Appendix S1. Supporting Information. [file PDS-34-e70178-s001.docx]

**Appendix A: Search Strategy**

**Table 1: Initial MEDLINE search including 1946 to September 20, 2023**

| # | Query | Results |
| --- | --- | --- |
| 1 | pharmaceutical preparations/ or controlled substances/ or exp dosage forms/ or exp drug combinations/ or drugs, essential/ or drugs, generic/ or exp nonprescription drugs/ or pharmaceutical preparations, dental/ or prescription drugs/ or prodrugs/ or exp solutions/ or synthetic drugs/ or exp "pharmacological actions (non mesh)"/ or drug industry/ or drug therapy/ | 5,596,503 |
| 2 | sd.fs. | 71,200 |
| 3 | 1 and 2 | 10,718 |
| 4 | (shortage* or discontinuation* or supply disruption* or recall* or stock out*).tw,kf. | 202,311 |
| 5 | 3 and 4 | 1,095 |
| 6 | drug recalls/ or safety-based drug withdrawals/ | 567 |
| 7 | ((drug or drugs or medicine or medication* or vaccin*) adj5 (shortage* or recall* or stock out* or supply disruption* or market withdrawal* or market discontinuation*)).tw,kf. | 4,140 |
| 8 | ((safety or regulat*) adj3 (drug withdrawal or medication withdrawal)).tw,kf. | 24 |
| 9 | (discont* adj3 (drug or drugs or medication* or medicine) adj10 (safety or regulat*)).tw,kf. | 173 |
| 10 | 5 or 6 or 7 or 8 or 9 | 5,355 |
| 11 | drug utilization/ or exp prescriptions/ or drug administration schedule/ or exp drug prescriptions/ | 163,912 |
| 12 | ((drug or drugs or medication* or medicine) adj3 utilizat*).tw,kf. | 6,152 |
| 13 | utilization trend*.tw,kf. | 557 |
| 14 | (prescribing adj3 pattern*).tw,kf. | 4,823 |
| 15 | practice patterns, pharmacists'/ or practice patterns, dentists'/ or practice patterns, physicians'/ | 69,611 |
| 16 | ((medication* or medicine or drug or drugs) adj3 dispensing pattern*).tw,kf. | 33 |
| 17 | prescription*.tw,kf. | 128,802 |
| 18 | (utilization pattern* or "pattern* of utilization" or treatment pattern* or "drug use").tw,kf. | 65,166 |
| 19 | or/11-18 | 381,740 |
| 20 | 10 and 19 | 636 |

**Table 2: Initial EMBASE search including 1947 to September 20, 2023**

| # | Query | Results |
| --- | --- | --- |
| 1 | drug/ or behind the counter drug/ or controlled substance/ or essential drug/ or generic drug/ or non prescription drug/ or prescription drug/ or drug combination/ or prodrug/ or exp drug dosage form/ or "solution and solubility"/ or drug industry/ | 981,822 |
| 2 | (shortage* or discontinuation* or supply disruption* or recall* or stock out*).tw,kf. | 307,319 |
| 3 | 1 and 2 | 15,483 |
| 4 | drug recall/ or product recall/ or drug shortage/ or medical resource shortage/ | 3,085 |
| 5 | ((drug or drugs or medicine or medication* or vaccin*) adj5 (shortage* or recall* or stock out* or supply disruption* or market withdrawal* or market discontinuation*)).tw,kf. | 5,951 |
| 6 | ((safety or regulat*) adj3 (drug withdrawal or medication withdrawal)).tw,kf. | 32 |
| 7 | (discont* adj3 (drug or drugs or medication* or medicine) adj10 (safety or regulat*)).tw,kf. | 384 |
| 8 | 3 or 4 or 5 or 6 or 7 | 22,881 |
| 9 | "drug use"/ or drug utilization/ or prescription/ or drug administration/ or prescribing practice/ or clinical practice/ or pharmacy practice/ | 792,198 |
| 10 | ((drug or drugs or medication* or medicine) adj3 utilizat*).tw,kf. | 12,285 |
| 11 | utilization trend*.tw,kf. | 1,072 |
| 12 | (prescribing adj3 pattern*).tw,kf. | 8,773 |
| 13 | ((medication* or medicine or drug or drugs) adj3 dispensing pattern*).tw,kf. | 46 |
| 14 | prescription*.tw,kf. | 219,274 |
| 15 | (utilization pattern* or "pattern* of utilization" or treatment pattern* or "drug use").tw,kf. | 99,828 |
| 16 | 9 or 10 or 11 or 12 or 13 or 14 or 15 | 930,984 |
| 17 | 8 and 16 | 3,198 |

**Table 3: Updated MEDLINE search including 1946 to September 17, 2024**

| # | Query | Results |
| --- | --- | --- |
| 1 | pharmaceutical preparations/ or controlled substances/ or exp dosage forms/ or exp drug combinations/ or drugs, essential/ or drugs, generic/ or exp nonprescription drugs/ or pharmaceutical preparations, dental/ or prescription drugs/ or prodrugs/ or exp solutions/ or synthetic drugs/ or exp "pharmacological actions (non mesh)"/ or drug industry/ or drug therapy/ | 5,769,069 |
| 2 | sd.fs. | 71,827 |
| 3 | 1 and 2 | 10,868 |
| 4 | (shortage* or discontinuation* or supply disruption* or recall* or stock out*).tw,kf. | 218,814 |
| 5 | (supply chain* adj2 (issue* or problem* or disrupt* or breakdown or obstruct*)).tw,kf. | 539 |
| 6 | 3 and 4 | 1,131 |
| 7 | 3 and 5 | 7 |
| 8 | drug recalls/ or safety-based drug withdrawals/ | 578 |
| 9 | ((drug or drugs or medicine or medication* or vaccin*) adj5 (shortage* or recall* or stock out* or supply disruption* or market withdrawal* or market discontinuation* or supply chain issue* or supply problem* or supply chain problem* or supply obstruction* or supply chain obstruction* or supply issue*)).tw,kf. | 4,612 |
| 10 | ((safety or regulat*) adj3 (drug withdrawal or medication withdrawal)).tw,kf. | 26 |
| 11 | (discont* adj3 (drug or drugs or medication* or medicine) adj10 (safety or regulat*)).tw,kf. | 181 |
| 12 | 6 or 7 or 8 or 9 or 10 or 11 | 5,858 |
| 13 | drug utilization/ or exp prescriptions/ or drug administration schedule/ or exp drug prescriptions/ | 165,644 |
| 14 | ((drug or drugs or medication* or medicine) adj3 utilizat*).tw,kf. | 6,707 |
| 15 | utilization trend*.tw,kf. | 615 |
| 16 | (prescribing adj3 pattern*).tw,kf. | 5,157 |
| 17 | practice patterns, pharmacists'/ or practice patterns, dentists'/ or practice patterns, physicians'/ | 71,159 |
| 18 | ((medication* or medicine or drug or drugs) adj3 dispensing pattern*).tw,kf. | 34 |
| 19 | prescription*.tw,kf. | 138,251 |
| 20 | (utilization pattern* or "pattern* of utilization" or treatment pattern* or "drug use").tw,kf. | 68,962 |
| 21 | ((medication* or medicine or drug or drugs) adj3 purchas*).tw,kf. | 2,006 |
| 22 | or/13-21 | 397,891 |
| 23 | 12 and 22 | 688 |

**Table 4: Updated EMBASE search including 1947 to September 17, 2024**

| # | Query | Results |
| --- | --- | --- |
| 1 | drug/ or behind the counter drug/ or controlled substance/ or essential drug/ or generic drug/ or non prescription drug/ or prescription drug/ or drug combination/ or prodrug/ or exp drug dosage form/ or "solution and solubility"/ or drug industry/ | 1,056,712 |
| 2 | (shortage* or discontinuation* or supply disruption* or recall* or stock out*).tw,kf. | 333,231 |
| 3 | (supply chain* adj2 (issue* or problem* or disrupt* or breakdown or obstruct*)).tw,kf. | 503 |
| 4 | 1 and 2 | 17,551 |
| 5 | 1 and 3 | 54 |
| 6 | drug recall/ or product recall/ or drug shortage/ or medical resource shortage/ | 3,763 |
| 7 | ((drug or drugs or medicine or medication* or vaccin*) adj5 (shortage* or recall* or stock out* or supply disruption* or market withdrawal* or market discontinuation* or supply chain issue* or supply problem* or supply chain problem* or supply obstruction* or supply chain obstruction* or supply issue*)).tw,kf. | 6,622 |
| 8 | ((safety or regulat*) adj3 (drug withdrawal or medication withdrawal)).tw,kf. | 34 |
| 9 | (discont* adj3 (drug or drugs or medication* or medicine) adj10 (safety or regulat*)).tw,kf. | 413 |
| 10 | 4 or 5 or 6 or 7 or 8 or 9 | 26,006 |
| 11 | "drug use"/ or drug utilization/ or prescription/ or drug administration/ or prescribing practice/ or clinical practice/ or pharmacy practice/ | 854,566 |
| 12 | ((drug or drugs or medication* or medicine) adj3 utilizat*).tw,kf. | 13,233 |
| 13 | utilization trend*.tw,kf. | 1,187 |
| 14 | (prescribing adj3 pattern*).tw,kf. | 9,527 |
| 15 | ((medication* or medicine or drug or drugs) adj3 dispensing pattern*).tw,kf. | 47 |
| 16 | prescription*.tw,kf. | 236,219 |
| 17 | (utilization pattern* or "pattern* of utilization" or treatment pattern* or "drug use").tw,kf. | 106,638 |
| 18 | ((medication* or medicine or drug or drugs) adj3 purchas*).tw,kf. | 3,015 |
| 19 | 11 or 12 or 13 or 14 or 15 or 16 or 17 or 18 | 1,004,019 |
| 20 | 10 and 19 | 3,704 |

**Appendix B: Grey Literature Search Documentation**

**Table 1: Targeted website search**

| **Date** | **Organization & URL** | **Search Strategy** | **Number of Items Retrieved** | **Number of Items Included** |
| --- | --- | --- | --- | --- |
| May 1, 2024 | CADTH  https://www.cadth.ca/ | For all websites:  Used Advanced Google site/domain search and filtered pdf files for exact phrases:  1) drug shortage  2) medicine shortage  All items retrieved in with each search were reviewed for relevance by 1 reviewer. | 94 | 0 |
|  | Health Quality Ontario  https://www.hqontario.ca/Evidence-to-Improve-Care/Health-Technology-Assessment |  | 3 | 0 |
|  | Health Canada  https://www.canada.ca/en/health-canada.html |  | 208 | 1 |
|  | Canadian Institute for Health Information  https://www.cihi.ca/en |  | 1 | 0 |
|  | National Institute for Health and Care Excellence  https://www.nice.org.uk/guidance |  | 3 | 0 |
|  | Government of Canada  https://publications.gc.ca/site/eng/home.html |  | 111 | 1 |
|  | Canadian Pharmacists Association (CPA)  https://www.pharmacists.ca/ |  | 74 | 0 |
|  | US Food and Drug Administration (FDA)  https://www.fda.gov/ |  | 628 | 0 |
|  | American Society of Health System Pharmacists (ASHP)  https://www.ashp.org/ |  | 272 | 0 |
|  | RAND Corporation  https://www.rand.org/ |  | 4 | 2 |
|  | Ministry of Health Ontario  https://www.ontario.ca/page/ministry-health |  | 2 | 0 |
|  | Ontario Health  https://www.ontariohealth.ca/ |  | 3 | 0 |
|  | Public Health Ontario  https://www.publichealthontario.ca/ |  | 0 | 0 |
|  | European Medicines Agency (EMA)  https://www.ema.europa.eu/en |  | 61 | 0 |
|  | Therapeutic Goods Administration (TGA) - Australia  https://www.tga.gov.au/ |  | 181 | 0 |
| May 2, 2024 | Organization for Economic Co-operation and Development (OECD)  https://www.oecd.org/health/ |  | 4 | 0 |
|  | International Pharmaceutical Federation (FIP)  https://www.fip.org/ |  | 74 | 0 |
|  | World Health Organization (WHO)  https://www.who.int/ |  | 162 | 0 |
| May 3, 2024 | U.S. Department of Health and Human Services  https://www.hhs.gov/ |  | 115 | 4 |
|  | Office of the Assistant Secretary for Planning and Evaluation (ASPE) |  | 9 | 2 |
| **Total of 10 reports were found and 5 duplicates were excluded, resulting in a total of 5 reports.** | | | | |

**Table 2: Grey literature database search**

| **Date** | **Database & URL** | **Search Strategy** | **Number of Items Retrieved** | **Number of Items Included** |
| --- | --- | --- | --- | --- |
| May 5, 2024 | World Health Organization (WHO) Data collections  https://www.who.int/data/collections | 1) drug shortage  2) medicine shortage  All items retrieved in with each search were reviewed for relevance by 1 reviewer. | 0 | 0 |
|  | Health Services Research Projects in Progress  https://collections.nlm.nih.gov/ |  | 1422 | 0 |
|  | New York Academy of Medicine’s Grey Literature Report  https://catalog.nyam.org/ |  | 9 | 0 |
|  | Trip Pro  https://www-tripdatabase-com.myaccess.library.utoronto.ca/ |  | 8356 | 0 |
|  | OAIster  https://oaister.on.worldcat.org/discovery |  | 2612 | 0 |
| Databases primarily included guidelines, patient education materials and published studies, so no relevant items were found. | | | | |

**Table 3: Search engine search**

| **Date** | **Search Engine** | **Search Strategy** | **Number of Items Retrieved** |
| --- | --- | --- | --- |
| May 3, 2024 | Google | 1) drug shortage and use  2) medicine shortage and use  First 10 pages were reviewed for relevance by 1 reviewer. | 0 |

**Appendix C: Characteristics of Included Studies**

**Table 1: Citations for Included Studies**

| **Study ID** | **Citation** |
| --- | --- |
| **Published Studies** | |
| 1 | Alpert A, Jacobson M. Impact of Oncology Drug Shortages on Chemotherapy Treatment. Clinical pharmacology and therapeutics. 2019;106(2):415-21. |
| 2 | Annema PA, Derijks HJ, Bouvy ML, van Marum RJ. Impact of Drug Recalls on Patients in The Netherlands: A 5-Year Retrospective Data Analysis. Clin Pharmacol Ther. 2024;115(6):1365-71. |
| 3 | Arinze JT, de Ridder MAJ, Vojinovic D, van Ballegooijen H, Markov E, Duarte-Salles T, et al. Drug Utilisation Patterns of Alternatives to Ranitidine-Containing Medicines in Patients Treated with Ranitidine: A Network Analysis of Data from Six European National Databases. Drug Saf. 2023;46(12):1353-62. |
| 4 | Barber KE, Bell AM, Travis King S, Parham JJ, Stover KR. Impact of piperacillin-tazobactam shortage on meropenem use: implications for antimicrobial stewardship programs. The Brazilian Journal of Infectious Diseases. 2016;20(6):631-4. |
| 5 | Barnes TA, Feng HX, Herrera NA, Leathers LA. The impact of intravenous opioid shortages on postoperative pain control in a pediatric cardiac intensive care unit. J Card Surg. 2020;35(4):840-4. |
| 6 | Becker DJ, Talwar S, Levy BP, Thorn M, Roitman J, Blum RH, et al. Impact of oncology drug shortages on patient therapy: unplanned treatment changes. J Oncol Pract. 2013;9(4):e122-8. |
| 7 | Bosso JA, Kokko H. Assessment of the Impact of an Antibiotic Shortage: Patient Outcomes and Costs. Hospital Pharmacy. 2004;39(1):43-7. |
| 8 | Brewster RC, Khazanchi R, Butler A, O'Meara D, Bagchi D, Michelson KA. The 2022 to 2023 Amoxicillin Shortage and Acute Otitis Media Treatment. Pediatrics. 2023;152(3). |
| 9 | Bykov K, Gagne JJ, Wang B, Choudhry NK. Impact of a Metoprolol Extended Release Shortage on Post-Myocardial Infarction beta-Blocker Utilization, Adherence, and Rehospitalization. Circulation Cardiovascular quality and outcomes. 2018;11(10):e004096. |
| 10 | Callaway Kim K, Rothenberger SD, Tadrous M, Hernandez I, Gellad WF, Devine JW, et al. Drug Shortages Prior to and During the COVID-19 Pandemic. JAMA Netw Open. 2024;7(4):e244246. |
| 11 | Choi Y, Santhireswaran A, Chu C, Suda KJ, Hernandez I, Magnani JW, et al. Effects of the July 2018 worldwide valsartan recall and shortage on global trends in antihypertensive medication use: a time-series analysis in 83 countries. BMJ Open. 2023;13(1):e068233. |
| 12 | Chun B, He M, Jones C, Vasan R, Gabriel N, Jacobs BL, et al. Variation in Statewide Intravesical Treatment Rates for Non-Muscle Invasive Bladder Cancer During the Bacillus Calmette-Guerin Drug Shortage. Urology. 2023;177(wsy, 0366151):74-80. |
| 13 | Desai RJ, Sarpatwari A, Gautam N, Lii J, Fischer MA, Gagne JJ. Changes in Utilization of Generic Angiotensin Receptor Blockers Following Product Recalls in the United States. JAMA. 2020;323(1):87-9. |
| 14 | Deutsch AJ, Gajdosik DA, Ramdin C, Tebo C, Mazer-Amirshahi M, Fox ER, et al. The Impact of the Parenteral Opioid Medication Shortages on Opioid Utilization Practices in the Emergency Department of Two University Hospitals. Journal of medical toxicology : official journal of the American College of Medical Toxicology. 2021;17(4):372-7. |
| 15 | Dorsey ER, Thompson JP, Dayoub EJ, George B, Saubermann LA, Holloway RG. Selegiline shortage: Causes and costs of a generic drug shortage. Neurology. 2009;73(3):213-7. |
| 16 | Eworuke E, Shinde M, Hou L, Paterson MJ, Jensen PB, Maro JC, et al. Valsartan, Losartan and Irbesartan use in the USA, UK, Canada and Denmark after the nitrosamine recalls: A descriptive cohort study. BMJ Open. 2023;13(4):e070985. |
| 17 | Fenna J, Chu C, Hassan R, Gomes T, Tadrous M. Extent of a valsartan drug shortage and its effect on antihypertensive drug use in the Canadian population: a national cross-sectional study. CMAJ Open. 2021;9(4):E1128-e33. |
| 18 | Frangoul H, Al-Jadiry MF, Shyr Y, Ye F, Shakhtour B, Al-Hadad SA. Shortage of chemotherapeutic agents in Iraq and outcome of childhood acute lymphocytic leukemia, 1990-2002. N Engl J Med. 2008;359(4):435-7. |
| 19 | Gross AE, Johannes RS, Gupta V, Tabak YP, Srinivasan A, Bleasdale SC. The Effect of a Piperacillin/Tazobactam Shortage on Antimicrobial Prescribing and Clostridium difficile Risk in 88 US Medical Centers. Clin Infect Dis. 2017;65(4):613-8. |
| 20 | Gunning R, Chu C, Nakhla N, Kim KC, Suda KJ, Tadrous M. Major Shifts in Acid Suppression Drug Utilization After the 2019 Ranitidine Recalls in Canada and United States. Dig Dis Sci. 2023;68(8):3259-67. |
| 21 | Haider A, Qian Y, Lu Z, Naqvi S, Zhuang A, Reddy A, et al. Implications of the Parenteral Opioid Shortage for Prescription Patterns and Pain Control Among Hospitalized Patients With Cancer Referred to Palliative Care. JAMA oncology. 2019;5(6):841-6. |
| 22 | Harbarth S, Gundlapalli AV, Stockdale W, Samore MH. Shortage of penicillin G: Impact on antibiotic prescribing at a US tertiary care centre. International Journal of Antimicrobial Agents. 2003;21(5):484-7. |
| 23 | Hayes MS, Ward MA, Slabaugh SL, Xu Y. Lessons from the leucovorin shortages between 2009 and 2012 in a medicare advantage population: where do we go from here? American health & drug benefits. 2014;7(5):264-70. |
| 24 | Hedenmalm K, Quinten C, Kurz X, Bradley M, Lee H, Eworuke E. A collaborative study of the impact of N-nitrosamines presence and ARB recall on ARB utilization - results from IQVIA TM Disease Analyzer Germany. European journal of clinical pharmacology. 2023;79(6):849-58. |
| 25 | Jackevicius CA, Krumholz HM, Chong A, Koh M, Ozaki AF, Austin PC, et al. Population Impact of Generic Valsartan Recall. Circulation. 2020;141(5):411-3. |
| 26 | Katsivalis KV, Kosacz J, Austin Szwak J. Opioid Use in Vaso-Occlusive Crisis During Intravenous Opioid Drug Shortage. Hospital pharmacy. 2022;57(6):721-6. |
| 27 | Khanna A, Yerram N, Zhu H, Kim S, Abouassaly R. Utilization of Bacillus Calmette-Guerin for Nonmuscle Invasive Bladder Cancer in an Era of Bacillus Calmette-Guerin Supply Shortages. Urology. 2019;124((Khanna, Yerram, Abouassaly) Glickman Urologic and Kidney Institute, Cleveland Clinic, Cleveland, OH, United States(Zhu, Abouassaly) Louis Stokes Cleveland VA Medical Center, Cleveland, OH, United States(Kim) University Hospitals Cleveland Medical Center):120-6. |
| 28 | Khumra S, Mahony AA, Devchand M, Walker ST, Garrett K, Grayson ML, et al. Counting the cost of critical antibiotic shortages. The Journal of antimicrobial chemotherapy. 2019;74(1):273-5. |
| 29 | King ST, Barber KE, Parham JJ, Stover KR. Shifts in antimicrobial consumption and infection rates before and during a piperacillin/tazobactam shortage. J Glob Antimicrob Resist. 2017;11:111-3. |
| 30 | Koizumi R, Kusama Y, Asai Y, Yoshiaki G, Muraki Y, Ohmagari N. Effects of the cefazolin shortage on the sales, cost, and appropriate use of other antimicrobials. BMC health services research. 2021;21(1):1118. |
| 31 | Konstantelos N, Tourchian N, McCormack D, Lecce P, Tadrous M, Gomes T. Impact of policy changes and drug shortages on acamprosate and naltrexone use in Ontario, Canada. Drug and alcohol dependence. 2023;242(ebs, 7513587):109705. |
| 32 | Ku M, Je NK. Prescription changes in patients with gastrointestinal disorders after withdrawal of ranitidine: a nationwide population-based cohort study. Current Medical Research and Opinion. 2023;39(2):197-203. |
| 33 | Ladha KS, Nanji KC, Pierce E, Poon KT, Hyder JA. The Impact of a Shortage of Pharmacy-Prepared Ephedrine Syringes on Intraoperative Medication Use. Anesthesia and analgesia. 2015;121(2):404-9. |
| 34 | Ledlie S, Tadrous M, McCormack D, Campbell T, Leece P, Kleinman RA, et al. Assessing the impact of the slow-release oral morphine drug shortages in Ontario, Canada: A population-based time series analysis. International Journal of Drug Policy. 2023;118:104119. |
| 35 | Li H, Cimino SK. Clinical impact of the etoposide injection shortage. Journal of Oncology Pharmacy Practice. 2020;26(1):187-92. |
| 36 | Maruyama K, Sekiya K, Yanagida N, Yasuda S, Fukumoto D, Hosoya S, et al. The impact of meropenem shortage and post-prescription review and feedback on broad-spectrum antimicrobial use: An interrupted time-series analysis. Infect Prev Pract. 2024;6(3):100380. |
| 37 | McAlister FA, Youngson E. Impact of the Generic Valsartan Recall in Alberta, Canada. Journal of the American College of Cardiology. 2020;75(15):1860-2. |
| 38 | Mendez MN, Gibbs L, Jacobs RA, McCulloch CE, Winston L, Guglielmo BJ. Impact of a piperacillin-tazobactam shortage on antimicrobial prescribing and the rate of vancomycin-resistant enterococci and Clostridium difficile infections. Pharmacotherapy. 2006;26(1):61-7. |
| 39 | Morizio PL, Britnell SR, Ottman AA. Impact of national valsartan recalls on Veterans' outcomes. Therapeutic Advances in Drug Safety. 2021;12((Morizio) Durham Veterans Affairs Health Care System, 508 Fulton St, Durham, NC 27705-3875, United States(Britnell, Ottman) Durham Veterans Affairs Health Care System, Durham, NC, United States). |
| 40 | N'Kaoua E, Attarian S, Delmont E, Campana-Salort E, Verschueren A, Grapperon AM, et al. Immunoglobulin shortage: Practice modifications and clinical outcomes in a reference centre. Revue neurologique. 2022;178(6):616-23. |
| 41 | Nagano H, Shin JH, Kunisawa S, Fushimi K, Nagao M, Imanaka Y. Impact of the cefazolin shortage on the selection and cost of parenteral antibiotics during the supply disruption period in Japan: A controlled interrupted time series analysis. Journal of Infection and Public Health. 2023;16(3):467-73. |
| 42 | Park SC, Gillis-Crouch GR, Cox HL, Donohue L, Morse R, Vegesana K, et al. Consecutive antibiotic shortages highlight discrepancies between microbiology and prescribing practices for intraabdominal infections. Antimicrobial Agents and Chemotherapy. 2021;65(5):e01980-20. |
| 43 | Pendergrast JM, Sher GD, Callum JL. Changes in intravenous immunoglobulin prescribing patterns during a period of severe product shortages, 1995-2000. Vox Sanguinis. 2005;89(3):150-60. |
| 44 | Petersen J, Freedman J, Ford L, Gawthrop M, Simons H, Edelstein M, et al. Changes to country-specific hepatitis A travel vaccination recommendation for UK travellers in 2017-responding to a vaccine shortage in the national context. Public health. 2019;168(qi7, 0376507):150-6. |
| 45 | Phakey S, Shen A. Impact of semaglutide and dulaglutide shortages on Pharmaceutical Benefits Scheme prescriptions supplied for type 2 diabetes treatment. Aust J Gen Pract. 2024;53(1-2):57-61. |
| 46 | Pluss-Suard C, Pannatier A, Ruffieux C, Kronenberg A, Muhlemann K, Zanetti G. Changes in the use of broad-spectrum antibiotics after cefepime shortage: a time series analysis. Antimicrobial agents and chemotherapy. 2012;56(2):989-94. |
| 47 | Roberts R, Ruthazer R, Chi A, Grover A, Newman M, Bhat S, et al. Impact of a national propofol shortage on duration of mechanical ventilation at an academic medical center. Critical care medicine. 2012;40(2):406-11. |
| 48 | Rodriguez-Monguio R, Lun Z, Bongiovanni T, Chen CL, Seoane-Vazquez E. Postoperative Respiratory Events in Surgical Patients Exposed to Opioid Analgesic Shortages Compared to Fully Matched Patients Non-exposed to Shortages. Drug safety. 2022;45(4):359-67. |
| 49 | Romito B, Stone J, Ning N, Yin C, Llano EM, Liu J, et al. How drug shortages affect clinical care: The case of the surgical anesthetic propofol. Hospital Pharmacy. 2015;50(9):798-805. |
| 50 | Rudolph UM, Enners S, Kieble M, Mahfoud F, Bohm M, Laufs U, et al. Impact of angiotensin receptor blocker product recalls on antihypertensive prescribing in Germany. Journal of human hypertension. 2021;35(10):903-11. |
| 51 | Santhireswaran A, Chu C, Kim KC, Gaudette É, Burry L, Clement F, et al. Early observations of Tier-3 drug shortages on purchasing trends across Canada: A cross-sectional analysis of 3 case-example drugs. PLoS One. 2023;18(12):e0293497. |
| 52 | Schade CP, Hannah KL. Impact of the 2004 influenza vaccine shortage on repeat immunization rates. Annals of Family Medicine. 2006;4(6):541-7. |
| 53 | Sheldon RJG, Pereira M, Aldersley G, Sales T, Hewitt J, Lyon R, et al. Clinical outcomes following switching antipsychotic treatment due to market withdrawal: a retrospective naturalistic cohort study of pipotiazine palmitate injection (Piportil Depot) discontinuation, subsequent acute care use and effectiveness of medication. Therapeutic Advances in Psychopharmacology. 2022;12. |
| 54 | Uda A, Onuma K, Shigemura K, Kitagawa K, Yan Y, Osawa K, et al. Impact of Cefazolin Shortage on Clinical Outcomes of Adult Patients with Bacteremia Caused by Methicillin-Susceptible Staphylococcus aureus in a Tertiary Care University Hospital. Antibiotics (Basel). 2021;10(10). |
| 55 | Vail E, Gershengorn HB, Hua M, Walkey AJ, Rubenfeld G, Wunsch H. Association Between US Norepinephrine Shortage and Mortality Among Patients With Septic Shock. Jama. 2017;317(14):1433-42. |
| **Grey Literature Reports** | |
| 56 | Patented Medicine Prices Review Board. Drug Shortages in Canada and their Impact on Public Drug Plans. 2022 [Available from: http://www.pmprb-cepmb.gc.ca/CMFiles/NPDUIS/refdocs/Reference%20Doc_NPDUIS%20Source%20Materials_EN.pdf. |
| 57 | Haninger K, Jessup A, Koehler K. Economic Analysis of the Causes of Drug Shortages. The Office of the Assistant Secretary for Planning and Evaluation (ASPE) At the U.S. Department of Health and Human Services, editor. Washington, DC. 2011. |
| 58 | Mulcahy A, Levin J, Shariq Qureshi N, Shetty K, Chen P, et al. Impact of Drug Shortages on Consumer Costs. RAND Corporation, editor. 2023. |
| 59 | Beleche T, Adetunji O, Parasrampurai S, Sen N, Sha S, Girouard E, et al. Impact of Drug Shortages on Patients in the United States: A Case Study of Three Drugs. Office of the Assistant Secretary for Planning and Evaluation of the U.S. Department of Health & Human Services. Washington, DC. 2024. |
| 60 | Mulcahy A, Rao P, Kareddy V, Agniel D, Levin J, Schwam D. Assessing Relationships Between Drug Shortages in the United States and Other Countries. RAND Corporation. 2021. |

**Table 2: Characteristics of included studies (N=43)**

| **Study ID** | **Country** | **Study Design** | **Objective(s)** | **Data Sources** | **Setting** |
| --- | --- | --- | --- | --- | --- |
| **Published Studies** | | | | | |
| 1 | United States | Repeated cross-sectional study | To provide a more general analysis of the impact of oncology drug shortages on outpatient chemotherapy treatment. | Surveillance, Epidemiology, and End Results (SEER) Cancer Registries, & Medicare | Outpatient Claims data |
| 2 | Netherlands | Repeated cross-sectional study | To create an overview of drug recalls that affected patients in the Netherlands and to establish the impact on patients. | Dutch Foundation for Pharmaceutical Statistics for dispensing data, and Dutch Health and Youth Care Inspectorate (IGJ), the Dutch Medicines Evaluation Board (MEB), and the European Medicines Agency (EMA) for recalls | Outpatient prescription dispensing data |
| 3 | Germany | Retrospective cohort study | To study drug utilization patterns of ranitidine and report discernible trends in treatment discontinuation and switching to alternative medications | Germany (IQVIA Disease Analyser [DA]), France (IQVIA Longitudinal Patient Database [LPD]), the United Kingdom (UK) (IQVIA Medical Research Data [IMRD]), the Netherlands (Integrated Primary Care Information [IPCI]), Belgium (IQVIA Longitudinal Patient Database [LPD]), and Spain (Information System for Research in Primary Care [SIDIAP]). | Inpatient & outpatient prescription dispensing data |
| 4 | United States | Retrospective cohort study | To evaluate the effect of a piperacillin-tazobactam shortage on meropenem use, related costs, and associated changes in antimicrobial stewardship activity | Chart review at large academic medical center | Inpatient drug administration data |
| 5 | United States | Retrospective chart review | To determine if a difference in postoperative pain scores in the period before and during the national intravenous opioid shortage exists, analyzed by differences in Face, Legs, Activity, Cry, Consolability (FLACC) scores. | Chart review at children's hospital | Inpatient drug administration data |
| 6 | United States | Retrospective cohort study | To examine outpatient oncology medication use at a single New York City university hospital between April and September 2010, and between April and September 2011 to evaluate changes in medication use influenced by chemotherapy drug shortages | Chart review at Continuum Cancer Center at Roosevelt Hospital | Outpatient drug administration data |
| 7 | United States | Retrospective cohort study | To elucidate the consequences of the piperacillin/tazobactam shortage in terms of alternatives used, overall cost of antimicrobial therapy, and patient outcomes, as reflected by length of stay and in-house mortality. | Chart review at 600-bed tertiary care teaching institution | Inpatient drug administration data |
| 8 | United States | Retrospective cohort study | To characterize the causal impact of an unexpected shock to high-dose amoxicillin availability on use of broader-spectrum antibiotic use. | Chart review at an urban freestanding children's hospital and affliate community center | Inpatient & outpatient prescription data |
| 9 | United States | Repeated cross-sectional study | To assess the impact of the metoprolol ER shortage on post-MI beta-blocker use, longer-term adherence, and clinical outcomes in a large population of patients discharged after hospitalization for acute MI. | Optum Clinformatics Data Mart | Outpatient drug claims data |
| 10 | United States | Cross-sectional matched cohort study | To estimate the proportion of supply chain issue reports associated with drug shortages overall and with the COVID-19 pandemic | IQVIA MIDAS | Inpatient & outpatient wholesale purchasing data |
| 11 | Global - 83 countries | Repeated cross-sectional study | To investigate the effects of the July 2018 valsartan recall and subsequent shortage on global utilisation trends of valsartan, angiotensin receptor blockers (ARBs) and angiotensin-converting enzyme inhibitors (ACEIs) across 83 countries overall and based on their economic development status. | IQVIA MIDAS | Inpatient & outpatient wholesale purchasing data |
| 12 | United States | Repeated cross-sectional study | To describe the effect of the Bacillus Calmette-Geurin (BCG) drug shortage on intravesical treatment patterns for non-muscle invasive bladder cancer using Medicare data. | Medicare | Inpatient & outpatient drug claims data |
| 13 | United States | Retrospective cohort study | To evaluate whether recalls shifted the utilization of ARBs, individually and as a class, evaluate dispensing patterns for recalled national drug codes to estimate the size of the patient population with potential exposure to impurities. | Optum Clinformatics Data Mart | Inpatient & outpatient drug claims data |
| 14 | United States | Retrospective review | To investigate the change in analgesic practice-related to a period of parenteral opioid shortage at two large urban, academic medical centers. | Chart Review of emergency department of the Hospital of the University of Pennsylvania and University Hospital (Newark) | Inpatient drug order data |
| 15 | United States | Repeated cross-sectional study | To evaluate prescription trends of generic selegiline and alternative monoamine oxidase type B inhibitors before and during the shortage and 2) quantify the economic impact of any resulting drug substitution. | IMS Health National Prescription Audit prescription data, & Medicare Prescription Drug Plan Finder | Inpatient & outpatient prescription data |
| 16 | USA, UK, Canada, Denmark | Retrospective cohort study | To examine valsartan, losartan and irbesartan usage and switching patterns in the USA, UK, Canada and Denmark before and after July 2018, when the first ARB (valsartan) was recalled. | US: FDA‚ Sentinel System; Canadian provinces of Manitoba, Nova Scotia, Ontario and Saskatchewan: Canadian Network for Observational Drug Effects (CNODES); Denmark: Danish National Prescription Registry (DNPR); UK: National Patient Register and the Clinical Practice Research Datalink (CPRD) | Inpatient & outpatient prescription dispensing data |
| 17 | Canada | Repeated cross-sectional study | To investigate the effects of the recall and shortage on use of valsartan, as well as other antihypertensive drugs, in Canada. | IQVIA Geographic Prescripion Monitor dataset | Outpatient prescription dispensing data |
| 18 | Iraq | Retrospective cohort study | To determine the effect of the shortage of chemotherapeutic agents on the outcome of acute lymphocytic leukemia in Iraqi children during the period of UN sanctions | Chart review at children's welfare teaching hospital | Inpatient administration data |
| 19 | United States | Retrospective cohort study | To identify any associations between changes in  antibiotic prescribing during the piperacillin/tazobactam shortage and hospital-onset C. difficile infection risk in a large collection of US medical centers. | MedMined | Inpatient drug order data |
| 20 | Canada and United States | Repeated cross-sectional study | To determine the impact of the ranitidine shortage on the utilization of histamine-2-receptor blockers (H2RAs) and proton pump inhibitors (PPIs) in Canada and the US. | IQVIA MIDAS | Inpatient & outpatient wholesale purchasing data |
| 21 | United States | Retrospective cohort study | To compare changes in opioid prescriptions and clinically improved pain (CIP) among patients treated by an inpatient palliative care (PC) team before and after our institution first reported the parenteral opioid shortage. | Chart review at the University of Texas MD Anderson Cancer Center | Inpatient & outpatient prescription data |
| 22 | United States | Retrospective cohort study | To describe temporal trends in penicillin G usage and indications at a US tertiary care centre from 1995 through 2000, thereby assessing the effect of the recent shortage of penicillin G in the US. | Chart review of US tertiary hospital in Salt Lake, Utah | Inpatient & outpatient prescription data |
| 23 | United States | Retrospective cohort study | To calculate the impact of the leucovorin shortages on primary treatment costs to patients and a health plan, and to present strategies for health plans to deal with future drug shortages. | Humana Medicare Advantage prescription drug plan administrative claims database | Outpatient prescription claims data |
| 24 | Germany | Repeated cross-sectional study | To investigate the impact of recalls of ARB-containing medicines between 2018 and 2019 in Germany on prescribing of individual ARBs in relation to total ARB prescribing and on switching from one ARB to an alternative ARB or an ACEI. | IQVIA Disease Analyzer | Outpatient prescription data |
| 25 | Canada | Repeated cross-sectional study | To determine impact of valsartan recall on emergency department visits and hospitalization rates for hypertension, heart failure, myocardial infarction, stroke. | ICES - ODB, RPDB, CIHI, NACRS, Statistics Canada | Inpatient & outpatient prescription data |
| 26 | United States | Retrospective cohort study | To assess IV opioid medication utilization before and during the shortage. Secondary objectives included total opioid consumption, length of stay, and prescribing of non-opioid analgesic. | Chart review at University of Chicago Hospital | Inpatient drug prescription and administration data |
| 27 | United States | Retrospective cohort study | To study trends in BCG utilization for nonmuscle invasive bladder cancer before and during national BCG shortages. | National Cancer Database | Inpatient prescription data |
| 28 | Australia | Retrospective cohort study | To evaluate the impact of piperacillin/tazobactam and gentamicin shortages on hospital antibiotic use and associated costs. | Chart review at Austin Health teaching hospital | Inpatient prescription data |
| 29 | United States | Retrospective cohort study | To assess the impact of complete shortage of piperacillin/tazobactam on the Gram-negative-active antimicrobial prescribing trends and hospital-acquired infection rates in the immediate peri-shortage period. | Chart review at University Medical Center | Inpatient drug administration data |
| 30 | Japan | Retrospective cohort study | To evaluate the effects of the serious cefazolin shortage in 2019 in Japan on the sales, costs, and appropriate use of other antimicrobials. | IQVIA Japan | Inpatient & outpatient wholesale purchasing data |
| 31 | Canada | Repeated cross-sectional study | To examine the impacts of expanded public drug coverage in 2018, and the subsequent acamprosate drug shortage in 2019 on utilization rates of these medications across Ontario, Canada. | ICES Ontario | Outpatient prescription dispensing data |
| 32 | South Korea | Retrospective cohort study | To investigate changes in the treatment regimen and their influencing factors after the ranitidine recall. | Health Insurance Review and Assessment Service-National Patient Sample | Outpatient prescription claims data |
| 33 | United States | Retrospective cohort study | To determine whether anesthesia providers changed their practice of medication administration in response to a shortage of pharmacy-pre-pared syringes of ephedrine. | Chart review at Massachusetts General Hospital | Inpatient drug administration data |
| 34 | Canada | Repeated cross-sectional study | To investigate the impact of slow-release oral morphine-24 drug shortages on patterns of treatment discontinuation in Ontario stratified by high and low dose slow-release oral morphine. | ICES Ontario | Outpatient prescription dispensing data |
| 35 | United States | Retrospective cohort study | To determine the percentage of patients who required a change in treatment due to the etoposide injection drug shortage. | Chart review at Pennsylvania Hospital | Inpatient prescription data |
| 36 | Japan | Retrospective cohort study | To assess the impact of the meropenem shortage and the post-prescription monitoring and feedback on broad-spectrum antimicrobial use and mortality. | Chart review at National Hospital Organization Sagamihara Hospital | Inpatient drug administration data |
| 37 | Canada | Retrospective cohort study | To examine the impact of the generic valsartan recall on patient treatment, outcomes, and health services use in the Canadian province of Alberta. | Alberta Pharmacy Information Network, Discharge Abstract Database, Alberta Practioner Claims database, National Ambulatory Care Reporting System database | Inpatient & outpatient prescription dispensing data |
| 38 | United States | Retrospective chart review | To evaluate the impact of a shortage of piperacillin-tazobactam in the United States in 2002 on antimicrobial prescribing and associated rates of vancomycin-resistant enterococci and C. difficile infections. | Chart review at University of California San Francisco (UCSF) Medical Center | Inpatient drug administration data |
| 39 | United States | Retrospective cohort study | To determine how the valsartan recall impacted clinical endpoints and subsequent prescribing of antihypertensives. | Chart review of tertiary Veterans Affair health care system | Inpatient & outpatient prescription data |
| 40 | France | Retrospective cohort study | To evaluate the impact of the intravenous immunoglobulins shortage on patient care in one French referral centre considering practice modifications and clinical impact. | AXIGATE university center database and chart review | Inpatient prescription data |
| 41 | Japan | Repeated cross-sectional study | To compare the proportion of each parenteral antibiotic use before and during the supply disruption period and investigate the impact of the cefazolin shortage on the prescription pattern and cost of parenteral antibiotic use for inpatients in hospitals classified by their susceptibility to the cefazolin supply disruption. | Diagnosis Procedure Combination (DPC) database | Inpatient prescription data |
| 42 | United States | Retrospective cohort study | To characterize changes in antimicrobial prescribing and microbiology in patients with intra-abdominal infection during shortages of piperacillin/tazobactam and cefepime. | University of Virginia Clinical Data Repository and Infection Prevention and Control Database | Inpatient prescription data |
| 43 | Canada | Retrospective cohort study | To identify the disease conditions for which intravenous immunoglobulin was being prescribed in academic hospitals during this period, and to explore the effects that intravenous immunoglobulin shortages had on prescribing patterns. | Chart review at Toronto General Hospital, Toronto Western Hospital, Princess Margaret Hospital, Sunnybrook Hospital and blood bank record | Inpatient prescription data |
| 44 | Global - 67 countries | Retrospective cohort study | First, to document the review process for changing the recommendations for the UK travellers in June 2017. Second, to study the impact of these changes on prescribing in general practice in 2017 compared with the previous 5 years. Third, to study any changes in hepatitis A notifications in June-October 2017 compared with the previous 5 years. | National Health Service Business Service Authorities | Outpatient prescription data |
| 45 | Australia | Retrospective cohort study | To measure the impact of semaglutide and dulaglutide shortages on their use in type 2 diabetes treatment by examining trends in the actual and predicted number of Australian Pharmaceutical Benefits Scheme (PBS) and Repatriation PBS (RPBS) prescriptions supplied during 2021 and 2022. | Australian Pharmaceutical Benefits Scheme (PBS) and Repatriation PBS (RPBS) | Outpatient prescription data |
| 46 | Switzerland | Retrospective cohort study | To assess the impact of the 2007 cefepime shortage on the use and costs of alternative broad-spectrum antibiotics, on antibiotic policy, and on resistance of Pseudomonas aeruginosa toward carbapenems, ceftazidime, and piperacillin-tazobactam. | Swiss hospital pharmacy data | Inpatient prescription data |
| 47 | United States | Retrospective cohort study | To measure the impact of a national propofol shortage on the duration of mechanical ventilation. | Respiratory therapy ICU database at Tufts Medical Center | Inpatient drug administration data |
| 48 | United States | Retrospective cohort study | To assess perioperative opioid analgesic use and related postoperative hypoxemia (oxygen saturation less than 90%) in surgical patients exposed to prescription opioid shortages compared to propensity score-matched patients non-exposed to opioid shortages. | Chart review at the University of California San Francisco (UCSF) Medical Center and FDA | Inpatient prescription data |
| 49 | United States | Retrospective cohort study | To survey the clinical impact of the abrupt propofol shortage at our hospital and to survey for any change in perioperative mortality. | Chart review at University of Texas, Southwestern Medical Center | Inpatient drug administration data |
| 50 | Germany | Retrospective cohort study | To analyze the utilization of valsartan, all ARBs, and other alternative antihypertensive drugs, that are ACEI, beta-blockers (BB), and calcium channel blockers (CCB) before and after the recalls in Germany. | German Institute for Drug Use Evaluation (DAPI) | Outpatient prescription dispensing data |
| 51 | Canada | Repeated cross-sectional study | To describe drug purchasing trends in response to Tier-3 shortages using three case-examples of hydralazine, sarilumab and medroxyprogesterone acetate | IQVIA MIDAS | Inpatient & outpatient wholesale purchasing data |
| 52 | United States | Retrospective cohort study | To assess the impact of the severe influenza vaccine shortage of 2004 on individual physicians' immunization performance. | Medicare | Inpatient & outpatient drug claims data |
| 53 | United Kingdom | Retrospective cohort study | To identify a cohort of patients switching from pipotiazine following the 2015 pipotiazine palmitate withdrawal and explore factors associated with effectiveness of the medication switched to and subsequent acute service use. | Chart review at Sussex Partnership NHS Foundation Trust | Inpatient & outpatient prescription data |
| 54 | Japan | Retrospective cohort study | To evaluate the effect of a cefazolin shortage on the clinical outcomes of patients with bacteremia caused by methicillin-susceptible S. aureus at a university hospital | Chart review at Kobe University Hospital | Inpatient drug administration data |
| 55 | United States | Retrospective cohort study | To assess changes to patient care and outcomes associated with a 2011 national shortage of norepinephrine, the first-line vasopressor for septic shock. | Premier Healthcare Database | Inpatient drug administration data |
| **Grey Literature Reports** | | | | | |
| 56 | Canada | Retrospective cohort study | To examine trends in the number, market segment, therapeutic area, and duration of drug shortages in Canada and explores their impact on public drug plan beneficiaries as well as on public plan spending | Canadian Institute for Health Information’s (CIHI) National Prescription Drug Utilization Information System (NPDUIS) | Outpatient prescription data |
| 57 | United States | Retrospective cohort study | To examine volume of sales of oncology sterile injectables prior to shortages and after for drugs that did and did not experience a drug shortage | Medicare Part B data | Inpatient & outpatient purchasing and reimbursement data |
| 58 | United States | Retrospective cohort study | To describe consumer costs associated with drug shortages | IQVIA National Sales Perspective data and IQVIA Total Patient Tracker | Inpatient & outpatient wholesale purchasing and prescription dispensation data |
| 59 | United States | Retrospective cohort study | To examine trends in the utilization of the selected drugs used in the treatment of patients with cancer before, during, and after shortages, to examine whether there were differences in the unique number of patients taking the drug, the dosage associated with an administration, or the service charge | IQVIA Medical Claims Data | Outpatient claims data |
| 60 | Global - Canada, France, Germany, Italy, Japan, UK, US and Australia | Retrospective cohort study | To assess the extent to which shortages in the United States are associated with changes in volume, prices, and other outcomes in other countries | IQVIA MIDAS | Inpatient & outpatient wholesale purchasing data |

**Table 2: Shortage Characteristics**

| **Study ID** | **Shortage Drug(s)** | **Drug Class** | **Comparator Drugs** | **Shortage Start/End Dates** | **Type of Supply Issue & Reason** | **Covariates Measured** |
| --- | --- | --- | --- | --- | --- | --- |
| **Published Studies** | | | | | | |
| 1 | Doxorubicin, Fluorouracil, Oxaliplatin, Cytarabine, Decarbazine, Leuprolide  Oral & parenteral  Prescription | Antineoplastic | NA | 2004-2011 | Shortage  Reason not reported | NA |
| 2 | 35 drug recalls  Oral, parenteral, topical, opthalmic & inhalation  Prescription | NA | NA | NA | Recall  Product contamination, sterility issues, product physical issues, manufacturing lab controls, product label, product packaging, suspended registration | NA |
| 3 | Ranitidine  Oral  Prescription | Histamine II blocker | Other histamine H2RAs (including cimetidine, famotidine, nizatidine, niperotidine, roxatidine, lafutidine), PPIs, and other medicinal products for acid-related disorders | September 2019 | Recall  Detection of N-Nitrosodimethylamine (NDMA) impurity in active pharmaceutical ingredient (API) manufacturing plant | Age & sex |
| 4 | Opioids  Parenteral  Prescription | Antibiotic | Meropenem | January 2015 | Shortage  Reason not reported | Demographic characteristics, medication allergy information, length of stay, location (ICU vs ward), in-hospital mortality, dose, duration, and frequency of meropenem administration, comorbidities, indication for meropenem treatment, and adverse drug reactions |
| 5 | Opioids  Parenteral  Prescription | Opioids | Acetaminophen, nonsteroidal anti‐inflammatory drug (NSAID), epidural | January 2018 | Shortage  Reason not reported | Demographic information including age, weight, medication allergies, and primary diagnosis, as well as pain management regimen in morphine equivalents, acetaminophen and NSAID usage, epidural usage, FLACC scores, time until extubation, and intensive care unit (ICU) days |
| 6 | Antineoplastic agents  Parenteral  Prescription | Antineoplastic | NA | No specific shortage | Shortage  Increased demand | demographic data (age, sex, comorbidities) |
| 7 | Piperacillin/Tazobactam  Parenteral  Prescription | Antibiotic | Ampicillin/sulbactam, ticarcillin/clavulanate, cefepime, cefotaxime, cefotetan, ceftriaxone, clindamycin, ciprofloxacin, imipenem, and metronidazole | March 2002 - August 2002 | Shortage  Reason not reported | NA |
| 8 | Amoxicillin  Parenteral  Prescription | Antibiotic | Amoxicillin-clavulanate, cefdinir, or other antibiotics | October 2022 | Shortage  Reason not reported | Patient demographics (ethnicity, sex, sociodemographics) |
| 9 | Metoprolol extended release  Oral  Prescription | Beta-blocker | Unaffected metoprolol ER generics: metoprolol tartrate, acebutolol, atenolol, betaxolol, bisoprolol, arvedilol, labetalol, nadolol, penbutolol, propranolol, timolol | February 2009 - June 2010 | Shortage  Reason not reported | Patient baseline characteristics |
| 10 | All drugs with a supply chain issue between 2017-2021  Oral, parenteral, inhalation, ophthalmic & otic  Prescription | Many | Drugs without a supply chain issue | NA | Recall, discontinuation and shortages  Many reasons | Drug characteristics (formulation, age, essential medicine status, clinician vs self-administered, sales volume, number of manufacturers) |
| 11 | Valsartan  Oral  Prescription | Angiotensin II receptor blocker | Non-valsartan ARBs, ACEIs | July 2018 | Recall  Detection of NDMA impurity in API manufacturing plant | NA |
| 12 | Bacillus Calmette-Guerin  Parenteral  Prescription | Biologic immunomodulator | NA | July 2012 - December 2018 | Shortage  Detection of fungal contamination and manufacturer withdrawal from market | Patient index date, age, sex, race, rurality, and region of residence |
| 13 | Valsartan, losartan, irbesartan  Oral  Prescription | Angiotensin II receptor blocker | Non-recalled ARBs (olmesartan, telmisartan, candesartan, eprosatan) | Valsartan: July 2018, Irbesartan: October 2018, Losartan: November 2018 | Recall Detection of NDMA impurity in API manufacturing plant | NA |
| 14 | Opioids  Parenteral  Prescription | Opioids | Non-opioid medications and oral opioids | March - May 2018 | Shortage  Manufacturing interruptions including Hurricane Maria in Puerto Rico, governmental stipulations on production of medications, and changes in the pharmaceutical industry | NA |
| 15 | Generic selegiline  Oral  Prescription | Monoamine oxidase type B inhibitor | Branded capsules, orally disintegrating tablets, and rasagiline | September 2007 | Shortage  Increased demand | NA |
| 16 | Valsartan, Irbesartan, Losartan  Oral  Prescription | Angiotensin II receptor blocker | Non-recalled ARBs - azilsartan, candesartan, olmesartan, telimisartan | Valsartan: July 2018, Irbesartan: October 2018, Losartan: November 2018 | Recall  Detection of NDMA impurity in API manufacturing plant | Patient age, gender, race, clinical history |
| 17 | Valsartan  Oral  Prescription | Angiotensin II receptor blocker | Non-valsartan ARBs, ACEIs, other hypertensives | July 2018 | Recall  Detection of NDMA impurity in API manufacturing plant | NA |
| 18 | Antineoplastic agents  Oral & parenteral  Prescription | Antineoplastic | NA | 1990-2003 | Shortage  Political conflict | Age, white-cell count at presentation, hemoglobin level, presence or absence of organomegaly, and year of treatment |
| 19 | Piperacillin/Tazobactam  Parenteral  Prescription | Antibiotic | High *C. difficile* infection risk antibiotics - clindamycin; fluoroquinolones; carbapenems; second/third/fourth-generation cephalosporins aztreonam; and ampicillin/sulbactam | December 2014 | Shortage  Manufacturing difficulties | Hospital characteristics |
| 20 | Ranitidine  Oral  OTC & prescription | Histamine II blocker | Non-ranitidine H2RAs and PPIs | September 2019 | Recall  Detection of NDMA impurity in API manufacturing plant | NA |
| 21 | Opiods: morphine, hydromorphone, methadone and fentanyl  Parenteral  Prescription | Opioids | Non-parenteral opioids | February 2018 | Shortage  Increased demand, production delays, reductions in the production quotas, shortages of sterile injectable products | patient demographics (age, sex, marital status), route of administration, dose, tobacco use |
| 22 | Penicillin G  Parenteral  Prescription | Antibiotic | Ampicillin | October 1999 | Shortage  Abrupt cessation of production by major US manufacturer | Patient demographics |
| 23 | Leucovorin  Oral & parenteral  Prescription | Folic acid analog | Levoleucovorin | June 2010 | Shortage  Increased demand & manufacturing delays | NA |
| 24 | Valsartan, Losartan, Irbesartan  Oral  Prescription | Angiotensin II receptor blocker | Other ARBs (azilsartan, candesartan, eprosartan, olmesartan, telmisartan), ACEIs | July 2018 | Recall  Detection of NDMA impurity in API manufacturing plant | NA |
| 25 | Valsartan  Oral  Prescription | Angiotensin II receptor blocker | Other ARBs, ACEIs | July 2018 | Recall  Detection of NDMA impurity in API manufacturing plant | patient demographics (age, sex, comorbidities) |
| 26 | IV opioids  Parenteral  Prescription | Opioids | Oral opioids, other non-opioid analgesics | October 2017 | Shortage  Reason not reported | Non-rounding and rounding pharmacists |
| 27 | Bacillus Calmette-Geurin  Parenteral  Prescription | Biologic immunomodulator | NA | 2011 & 2012 | Shortage  Regulatory non-compliance, and batch contamination | Patient demographics, insurance coverage, tumor stage, facility volume |
| 28 | Piperacillin/Tazobactam and Gentamicin  Parenteral  Prescription | Antibiotic | Non-shortage antibiotics - ampicillin, cephalosporins, β-lactamase inhibitors, aminoglycosides, ciprofloxacin, clindamycin, metronidazole, meropenem, vancomycin | October 2017 - December 2017 | Shortage  Supply interruption | NA |
| 29 | Piperacillin/Tazobactam  Parenteral  Prescription | Antibiotic | Meropenem, cefepime, ceftriaxone, ceftazidime, ciprofloxacin and levofloxacin | January 2015 | Shortage  Reason not reported | NA |
| 30 | Cefazolin  Parenteral  Prescription | Antibiotic | Non-shortage antimicrobials – piperacillin/tazobactam, meropenem, ceftriaxone, vancomycin, clindamycin, ampicillin/sulbactam, levofloxacin, cefotiam, cefmetazole, flomoxef | January 2019 | Shortage  Manufacturing difficulties | NA |
| 31 | Acamprosate  Oral  Prescription | GABA receptor agonist | Naltrexone | January 2019 - June 2020 | Shortage  Broadened drug access | Demographic data |
| 32 | Ranitidine  Oral  OTC & prescription | Histamine II blocker | Other H2RAs (cimetidine, famotidine, nizatidine, lafutidine, and roxatidine), proton pump inhibitors (PPIs) (dexlansoprazole, esomeprazole, ilaprazole, lansoprazole, omeprazole, pantoprazole, rabeprazole, and s-pantoprazole), PCABs (revaprazan, tegoprazan, and vonoprazan), and a prostaglandin E1 analog (misoprostol) | September 2019 | Recall  Detection of NDMA impurity in API manufacturing plant | demographic data, other drug use, regions, type of medical institution, insurance type, other GI disorders |
| 33 | Ephedrine  Parenteral  Prescription | Anesthetic | phenylephrine | October 2012 | Shortage  Closure of pharmaceutical compounding company due to concerns of sterility-testing process | Patient demographics, provider type, surgery duration, type, airway type |
| 34 | Slow-release oral morphine - 24hr formulation (Kadian)  Oral  Prescription | Opioid agonist treatment | NA | November 2019, March 2020, March 2021, July 2021, August 2021 | Shortage  Manufacturing issues, increased demand, & shipping delays | Demographic data |
| 35 | Etoposide  Parenteral  Prescription | Antineoplastic | Etopophos injection, oral etoposide | January 2018 | Shortage  Increased demand, manufacturing delay & shortage of an active ingredient | Baseline data including age, gender, height, weight, dosing body surface area (BSA), serum creatinine, aspartate aminotransferase, alanine aminotransferase, cancer diagnosis, previous cancer treatments, current chemotherapy regimen, current cycle, drug formulation, and drug dose. |
| 36 | Meropenem  Parenteral  Prescription | Antibiotic | Tazobactam/piperacillin, cefepime, levofloxacin, and ciprofloxacin | September 2022 - February 2023 | Shortage  Manufacturing issues | NA |
| 37 | Valsartan  Oral  Prescription | Angiotensin II receptor blocker | Other angiotensin receptor blocker, brand-name valsartan | July 2018 | Recall  Detection of NDMA impurity in API manufacturing plant | Demographic data (age, sex, comorbidities) |
| 38 | Piperacillin/Tazobactam  Parenteral  Prescription | Antibiotic | Cephalosporins, fluoroquinolones, other B-lactamase inhibitors, carbapenams, aminoglycosides, clindamycin, metronidazole, vancomycin | March - August 2002 | Shortage  Manufacturing problems, distribution and ordering inefficiencies | Patient demographics, microbiologic reports |
| 39 | Valsartan, Irbesartan, Losartan  Oral  Prescription | Angiotensin II receptor blocker | Angiotensin-converting enzyme inhibitors, beta-blockers, calcium-channel blockers, mineralocorticoid receptor antagonist | March 2019 | Recall  Detection of NDMA impurity in API manufacturing plant | Demographic information (age, sex, race, comorbidities) |
| 40 | IV immunoglobulin  Parenteral  Prescription | Immunoglobulin | Plasma exchange, corticosteriods | July 2005 | Shortage  Increased demand & difficulty procuring raw materials | Demographics, disease progression |
| 41 | Cefazolin  Parenteral  Prescription | Antibiotic | Flomoxef, cefotiam, cefmetazole, ceftriaxone, ampicillin/sulbactam, clindamycin, vancomycin, and levofloxacin, piperacillin/tazobactam and meropenem | March - November 2019 | Shortage  Manufacturing difficulties | Hospital descriptive statistics (number of beds, teaching hospitals, eligible for other fees, etc.) |
| 42 | Piperacillin-tazobactam and Cefepime  Parenteral  Prescription | Antibiotic | Ciprofloxacin, metronidazole, ceftriaxone, meropenem, vancomycin | March 2015 | Shortage  Limited drug supply | Length of hospital stay, mortality, ICU transfer |
| 43 | Intravenous Immunoglobulin  Parenteral  Prescription | Immunoglobulin | NA | June 2005 | Shortage  Increased demand | Patient demographics (age, sex, weight) |
| 44 | Monovalent hepatitis A-containing vaccines  Parenteral  Prescription | Vaccine | NA | March 2017 | Shortage  Hepatitis A outbreak | NA |
| 45 | Semaglutide and Dulaglutide  Parenteral  Prescription | Glucagon-like peptide receptor antagonists | Predicted prescription numbers for shortage drugs | Semaglutide - April 2022, Dulaglutide - June 2022 | Shortage  Increased demand | NA |
| 46 | Cefepime  Parenteral  Prescription | Antibiotic | Ceftazidime, imipenem-cilastatin, meropenem, piperacillin-tazobactam | January - October 2007 | Market Withdrawal | NA |
| 47 | Propofol  Parenteral  Prescription | Anesthetic | Lorazepam, midazolam, and/or dexmedetomidine | October 2009 - January 2011 | Recall  Microbial contamination | Age, admission Acute Physiologic and Chronic Health Evaluation II score, admitting service (i.e., medical vs. surgical), presence at admission of the acute respiratory distress syndrome or sepsis (vs. other primary causes for ICU admission), presence of acute alcohol withdrawal at admission, the admission PaO2/FIO2 ratio, presence of renal failure (a serum creatinine 1.8 mg/dL at admission), and use of pressure-controlled ventilation as the most frequent mode of mechanical ventilation |
| 48 | Opioid  Oral & parenteral  Prescription | Opioids | NA | No specific shortage | Shortage  Reason not reported | Demographic data (age, insurance, sex,) and clinical characteristics (ASA score, pain scorem, substance use, etc.) |
| 49 | Propofol  Parenteral  Prescription | Anesthetic | Opioid and benzodiazepines, volatile anesthetic, methohexital, etomidate | August - October 2010 | Shortage  Poor drug availability | Simplified Acute Physiology Score II (SAPS II), Acute Physiology and Chronic Health Evaluation II (APACHE II), and Charlson Comorbidity Index (CCI) scores |
| 50 | Valsartan  Oral  Prescription | Angiotensin II receptor blocker | Other ARBs, ACEIs, beta-blockers, and calcium channel blockers | July 2018 | Recall  Detection of NDMA impurity in API manufacturing plant | NA |
| 51 | Hydralazine, sarilumab, and medroxyprogesterone acetate  Oral & parenteral  Prescription | Vasodilator, biologic, progestin | NA | Hydralazine: August 2019, Sarilumab: March 2020, Medroxy-progesterone: July 2021 | Shortage  Increase in demand, production delays, disruptions in drug manufacturing | NA |
| 52 | Influenza vaccine  Parenteral  Prescription | Vaccine | NA | June 2005 | Shortage  Manufacturing delay | Physician specialty, # of claims filed |
| 53 | Pipotiazine palmitate  Parenteral  Prescription | Antipsychotic | Clozapine, typical depot, atypical oral, atypical long-acting injectable | March 2015 | Market Withdrawal  Potential contamination issues | gender, age at switch, primary psychiatric diagnosis, duration of illness before switch, duration of pipotiazine depot treatment, 4 weekly pipotiazine dose, switch setting (inpatient or outpatient) and the antipsychotic the patient was switched to, if any |
| 54 | Cefazolin  Parenteral  Prescription | Antibiotic | Benzylpenicillin, ampicillin, ampicillin/sulbactam, piperacillin/tazobactam, cefotiam, meropenem vancomycin, daptomycin, and linezolid | March 2019 - January 2020 | Shortage  Problems with major supplier | Demographic data |
| 55 | Norepinephrine  Parenteral  Prescription | Vasopressor | Alternative vasopressors - phenylephrine, dopamine, epinephrine, vasopressin | February 2011 - February 2012 | Shortage  Manufacturing interruptions at 3 drug manufacturers | Patient demographics, comorbid diseases, acture organ dysfunction, race, surgical admissions |
| **Grey Literature Reports** | | | | | | |
| 56 | All Canadian drug shortages from 2017-2020  Oral, parenteral and other  Prescription | NA | NA | NA | Shortage  Reason not reported | Number of shortages, spending prior to shortage, number of beneficiaries prior to shortage, spending per beneficiary prior to shortage, shortage durations, number of DINs, formulations, branded, single-source drugs, patented medicines, therapeutic class |
| 57 | Oncology Sterile injectables  Parenteral  Prescription | Antineoplastic | Non-shortage oncology sterile injectables | 2008 | Shortage  Reason not reported | NA |
| 58 | All FDA-reported US drug shortages with available data, focused on case study drugs aspariginase, belatacept, carbidopa/levodopa, heparin, valsartan  Oral & parenteral  Prescription | Antineoplastic, immunosuppressant, anti-parkinson agent, anticoagulant, angiotensin-receptor blocker | NA | NA | Shortage  Reason not reported | Market structure, retail vs hospital dispensed, consumer age group and sex |
| 59 | Asparaginase, nelarabine, heparin  Oral & parenteral  Prescription | Antineoplastic, anticoagulant | Pegaspargase, cladribine, clofarabine, fludarabine, heparin, bivalirudin, lepirudin | Oct 2016 - Aug 2021, Oct 2018 - Jan 2020, Nov 2027 - ongoing | Shortage  Supply disruption, Increased demand, manufacturing delays | Patient demographics |
| 60 | All US drug shortages prior to or during March 2020  Oral, parenteral & other  Prescription | NA | NA | NA | Shortage  Reason not reported | Formulation, market type, manufacturer count, |

**Table 3: Changes in Drug Use**

| **Study ID** | **Relevant Outcomes** | **Statistical Analysis** | **Changes in Drug Use** |
| --- | --- | --- | --- |
| **Published Articles** | | | |
| 1 | Fraction of patients receiving treatment with each drug among those who were eligible for treatment during the shortage periods relative to the months before and after | Linear regression model | Decline in utilization by 4% for doxorubin and fluorouracil, 2.9% for oxaliplatin, and about 1% for cytarabine, dacarbazine and leuprolide |
| 2 | Total number of switches made by active drug users due to the drug recall | NA | An estimated 855,000 switches, where 292,000 patients were required to switch to a different brand of the same drug and 95,000 patients were required to switch to a different drug class. |
| 3 | Incident use of ranitidine, other H2RAs, and other alternative drugs during the pre-recall, during recall and post-recall periods; ranitidine discontinuation; and switching from ranitidine to alternative drugs | NA | Incidence rates declined to 0.3-3.8/1000 PY in the recall period from 0.8-9.0/1000 PY in the pre-recall period, and further dropped to 0-0.4/1000 PY in the post-recall period. |
| 4 | Meropenem utilization rates three months prior to shortage and three months during shortages | Chi-square test, Fisher’s exact test, Mann-Whitney U-test, Student's t-test | Meropenem usage increased by 11% during the shortage compared to pre-shortage periods |
| 5 | Morphine equivalents of IV pain medication used between shortage group and pre-shortage group | Descriptive statistics, t-test, quantile regression, Wilcoxon, Mann-whitney test, chi-square test | Morphine equivalents (ME) used decreased by 0.24 during the shortage period from 0.86 ME to 0.62 ME |
| 6 | Proportion of chemotherapy regimens changed as a result of drug shortages | Descriptive statistics, Fisher’s exact test | 9.8% of patients received alternative therapy due to shortages |
| 7 | Percent change in total daily doses (DDDs) per 1000 patient days in pre-shortage and post-shortage periods | Two-tailed Student’s t-test | Pip/tazo use decreased by 87.7% (50.0 DDD per 1000 PD to 6.1 DDD per 1000 PD), cefepime increased by 146.0%, ticarcillin/clavulanate increased by 296.9% |
| 8 | Odds of being prescribed amoxicillin | Logistic regression analysis | The odds of being prescribed amoxicillin decreased by 91%, and odds of being prescribed amoxicillin-clavulanate increased sevenfold and cefdinir increased ninefold |
| 9 | Monthly change in post-discharge beta-blocker use and monthly adherence from pre-shortage and post-shortage periods | Segmented linear regression | Monthly reduction in post-discharge beta-blocker use by 0.57% of patients and immediat edecrease in adherence by 4.58% days covered |
| 10 | The proportion of supply chain issues with a 33% decrease purchased united within 6 months of the supply chain issue report. | Logistic regression analysis | 13.7% of supply chain issue reports were associated with a 33% decrease in purchased units |
| 11 | Monthly purchasing of valsartan, ARBs and ACEIs in global context and by economic development level. Percent changes comparing pre-recall and post-recall periods compared with the same periods in the year prior | ARIMA modelling | Global valsartan utilization decreased by 15.7% |
| 12 | Rates of full induction therapy and type of induction therapy before and after the start of the shortage period and by year of diagnosis, and state-level changes in the proportion of patients initiating intravesical therapy with BCG before and after the start of the shortage period | ANOVA and chi-square for patient characteristics, no statistical model for drug use | BCG utilization decreased by 5.9% and propotion of patients that completed a full induction by 3.4%. 84% of reporting states had decreased BCG utilization ranging between 5% and 36% compared to pre-shortage rates |
| 13 | Monthly utilization between January 2018-March 2019 | NA | Valsartan use decreased by 11%, while losartan use decreased by 2% and irbestan use increased by 2% |
| 14 | Doses of opioids divided by number of ED visits for the site per month for periods before, during and after shortage. | Paired t-test & Wilcoxon signed rank test | Percentage of patients receiving opioids among ED visits decrease 4.3% post-shortage. IV opioid doses administered decreased 4.7% during the shortage and oral opioid doses decreased 3.1% during the shortage. |
| 15 | Monthly total prescriptions filled during and prior to shortage | Linear regression model | Prescriptions filled decreased by 11% during shortage |
| 16 | Monthly percentage of individual ARB usage and the number of specific ARB episodes, new ARB users and proportion of switching to non-index ARB, ACEI or CCB | Interrupted time series | 5 months after the recall, monthly proportions decreased by 10% in USA, 12% in Canada, 0.04% in Denmark and no changes in UK |
| 17 | Monthly prescriptions dispensed before and after shortage | ARIMA modelling | Valsartan use decreased 57.8%, from 362 231 prescriptions dispensed in June 2018 to 152892 in September 2018, use of non-valsartan ARBs increased 14.6%, from 1215278 to 1392699 prescriptions dispensed |
| 18 | Proportion of patients who did not receive complete treatment during 1990-1994 and 2000-2002 | Cox proportional hazard and multivariate analysis | During the shortage patients receiving less than 50% of prescribed chemotherapy was 20.1% in 1990-1994 to 54.3% in 2000-2002 |
| 19 | Usage of antibiotics in pre-shortage and post-shortage period for hospitals with severe shortage (>66%), moderate shortage (34-66%) and mild shortage (<33%), and used DOT per 1000 days at risk for evaluating drug use | Univariate analysis with Wilcoxon test | Hospitals with mild shortage had 8.8 decrease in DAR, moderate shortage hospitals had 41.9 decrease in DAR, and severe shortage hospitals had 67.2 decrease in DAR |
| 20 | Purchasing rate as monthly units purchased per 100,000 persons, and percent change in purchasing rates and 1, 3, 6, 12 and 24 months | ARIMA modelling | Purchasing rates decreased by 99% in Canada and 53% in US withing 1 month after the recalls |
| 21 | Opioid prescription patterns for month before and after shortage | Wilcoxon sum rank test | Prescribing decreased by 17% for the oncology team and 18% for the PC team |
| 22 | Total number of patient exposed to penicillin G each year from 1995-2000 | NA | Decreased use by 17 patients post-shortage |
| 23 | Percentage of patients receiving the drug in 2010 and 2011 | NA | Leucovorin use decreased by 15.8% from 2010 to 2011 |
| 24 | Monthly proportion of ARB prescriptions, quarterly proportion of incident ARB use, and switched from ARB to alternative ARB or ACE inhibitor | Interrupted time series | Proportion of valsartan prescriptions decreased by 18.1% and valsartan switching rate increased by 0.574 |
| 25 | Prescription patterns in recalled valsartan users before and after recall date | Segmented regression analysis | 82.6% switched to a non-valsartan ARB or a non-recalled valsartan product |
| 26 | Amount of opioid medication administered to patients before and during the shortage in morphine milligram equivalents between years | Chi-square and Wilcoxon sum rank test | 52% decrease in IV opioid use from 2017 to 2018, and oral opioid use increased by 28.9% |
| 27 | Rate of BCG utilization before and after supply interruption | Chi-square test, multivariate logistic regression and segmented regression | The rate of increase in BCG use decreased by 0.33 (2004-2012: +0.62% increase per year; 2013-2015: +0.29% increase per year) |
| 28 | Percent change in antibiotic use pre-shortage versus post-shortage | NA | 47% decrease in pip/tazo use, and 5% decrease in gentamicin use |
| 29 | Percent change and daily doses (DDD)/100 patient-days (PD) in antibiotic use pre-shortage versus post-shortage | Descriptive statistics | Pip/tazo use decreased by 95.2% during the shortage from 82.2% to 3.9%, meropenem and cefepime DDDs/1000 PDs increased 96.0% and 97.9%, respectively. Extended-spectrum cephalosporin use increased 30.1% for ceftriaxone and 94.2% for ceftazidime. |
| 30 | Monthly defined daily doses/1000 inhabitants/day (DID) from January 2016 to December 2019 | SARIMA Modelling | DID values sharply increased 1-month before the decrease, no numbers reported |
| 31 | Monthly dispensing rates of acamprosate and naltrexone per 100,000 ODB beneficiaries | ARIMA modelling | 98.1% reduction in acamprosate use but no impact to naltrexone use |
| 32 | Proportions of patients that switched to a an alternative drug after the recall | Chi-square test, Kaplan-Meier, and Cox proportional hazards regression analysis | 68.8% of patients switched from ranitidine to an alternative drug |
| 33 | Frequency of receiving atleast 1 bolus of ephedrine and phenylphrine, and total dosage administered | T-test, chi-square, and regression model | The administration of at least 1 bolus of ephedrine decreased significantly by 9.40% during the shortage compared to before |
| 34 | Changes in rate of discontinuation of slow-release oral morphine, and monthly percent changes in discontinuation | ARIMA modelling | Following the shortages in November 2019, March 2020, July 2021, and August 2021 there was an increase in discontinuations by 0.29%, 2.00%, 3.53% and 4.98%, respectively. |
| 35 | Proportion of patients that required change in treatment due to etoposide injection shortage | Fisher's exact test | 32% of patients switched treatment |
| 36 | Changes in slope for days of therapy (DOT) and days of antibiotic spectrum coverage (DASC) for broad-spectrum antimicrobials for the pre-shortage and shortage periods | Interrupted time series | Meropenem DOT decreased by -9.28 during shortage, cefepime increased by 11.84, and other antibiotics showed no significant changes |
| 37 | Proportion of patients the required to switch to another drug due to the shortage | Segmented regression analysis | 3 months after the recall, 13,368 of patients (91.1%) younger than 65 years had been switched to another drug compared with 18,688 (93.2%) of those 65 years or older |
| 38 | Number of charged doses and total grams for each anti-microbial for pre-shortage and during shortage period, a charged dose was defined as a dose of a drug billed to a patient account | Poisson regression analysis | Pip/tazo charged doses decrease by 67% from 2001 to 2002 |
| 39 | Number of patients with medications changed to other antihypertensives or titrations in the post-recall period | Paired t-test and McNemar's test | 11 patients were required to switch medication due to the recall |
| 40 | Number of treatment changes (decrease in IVIg dose, delay in IVIg treatment, discontinuation of IVIg treatment) for year prior and after shortage | T-test, and Wilcoxon signed rank test | 78% of patients had modifications to their IVIg treatment, 68% had a delya in treatment, 37% had a decrease in IVIg doses and 28% experienced treatment interruption |
| 41 | Propotion of drug us to the sum of all antibiotics during the shortage period to the year prior in shortage hospitals and non-shortage hospitals | Chi-square test and interrupted time series analysis | In shortage hospitals the proportion of cefazolin use to the sum of all selected antibiotics decreased by 12.4% during the shortage period, and the use of broader-spectrum beta-lactams and clindamycin increased - flomoxef up 58.1%, cefotiam up 63.1%, cefmetazole up 14.5%, ceftriaxone up 13.9%, and clindamycin up 20.1% |
| 42 | Days of therapy per 1000 patient-days for preshortage, TZP shortage, cefepime shortage and postshortage | Chi-square test and Kruskal-Wallis test | During the TZP shortage, there was a 93% reduction in TZP usage, a 190% increase in cefepime usage, a 57% increase in ceftriaxone usage, a 13% increase in ciprofloxacin usage, and a 74% increase in metronidazole usage compared to the preshortage period. During the cefepime shortage, there was a 69% reduction in cefepime usage relative to the preceding (TZP shortage) period; however, there was only a 9% reduction compared to the preshortage period, and cefepime usage was lowest in the postshortage period. |
| 43 | Annual number of patients treated with IVIg and annual amount if IVIg prescribed in kg | NA | After 1997 there was an abrupt decline in annual number of patients by 44, and the amount prescribed decreased by 7.4kg |
| 44 | Number of monthly prescriptions between January and September 2017 compared with the mean number of prescriptions for the same month in the previous 5 years | T-test | Mean number of prescriptions dropped by 23% in June 2017, 33% in July, 47% in August and 56% in September compared to the same months in the previous 5 years |
| 45 | Monthly changes in number of actual prescriptions, and relative difference between actual and predicted number of prescriptions | Holt-Winters forecast models | 17% decrease for semaglutide in March - Sept 2022 and 17% decrease for dulaglutide in August - September 2022, and 119,069 fewer semaglutide and 31,953 more dulaglutide prescriptions supplied than predicted in April-July and June- July 2022, respectively |
| 46 | Antibacterial consumption in grams converted into defined daily doses (DDD) and then expressed iDDD per 100 occupied bed days per month (DDD/100BD/month) for 3 groups: group A, without any cefepime supply since its withdrawal; group B, without any cefepime supply during the shortage and then supply of a generic as soon as it became available; and a control group C of hospitals with uninterrupted cefepime supply thanks to importation from abroad | Interrupted time series | Immediately after the withdrawal there was a decreasing trend in cefepime use at -0.95 in shortage hospitals, a decrease of -2.53 in group B, and no changes in control group C. |
| 47 | Proportion of patients receiving >24hrs of continuously infused sedative before shortage and after shortage period | Chi-square test, student's t-test, and Mann-Whitney U-test | The patients receiving >24hrs of continuous propofol infusion decreased by 84% after the shortage, lorazepam use increased by 8%, and midazolam use increase by 51% |
| 48 | Morphine milligram equivalents/day used by patient exposed to the shortage and not exposed to the shortage | Logistic regression analysis | Patients exposed to the shortage used 146.94 MME more or 1.25 times more than those not exposed to the shortage |
| 49 | Proportion of patients receiving propofol prior to shortage and etomidate post shortage | Chi-square test and unpaired t-test | Before the propofol shortage, 80% received propofol, and during the shortage, a 81% received etomidate. The use of etomidate increased by 600% after the propofol shortage. |
| 50 | Defined daily doses per 1000 insured persons (DID) on a daily basis in June and July 2018 compared to the year prior and monthly moving averages of DID for respective month and two previous months | NA | In the second quarter of 2019, valsartan use decreased by 64% from 39.0 to 14.2 DID, and by 57% in the fourth quarter to 16.9 DID. The use of alternative ARBs increase from 77.7 DID in the second quarter of 2018 to 121.9 DID by 57% in the fourth quarter of 2019. |
| 51 | Changes in drug purchasing at 1-, 3-, and 6-months after the onset of each Tier-3 drug shortage | ARIMA modelling | Medroxyprogesterone acetate experienced a significant shift (p = 0.0370) in purchasing following its shortage, and the 1-, 3-, and 6-month percent changes were +14.9%, +6.8% and -3.1%, respectively. Hydralazine and sarilumab did not show a significant shift. The 1-, 3-, and 6-month percent changes for hydralazine were +15.5%, +10.2%, and +9.6% respectively and +25.2%, +45.1% and +39.2 for sarilumab. |
| 52 | Physician continuity rate defined as proportion of patients receiving influenza immunization from a physician in 1 year who received a subsequent immunization from the same physician the subsequent year, other clinician rate defined as proportion of patients with claims from 1 physician in 1 year with a claim from another clinician the subsequent year, and vaccine claim trends by clinician | NA | Claims-based influenza vaccination rates declined by 14.1% in 2004, physician continuity rate declined by 51% in 2003-2004. The number of physicians filing 100 or more claims declined from 337 in 2003 to 130 in 2004. More than 25% of physicians had no repeat vaccinations of the same beneficiaries in 2004. |
| 53 | Number of patients that switched from pipotiazine due to market withdrawal | Logistic regression analysis and Kaplan-Meier | 137 of 205 patient switched to an alternative medication due to the market withdrawal |
| 54 | Proportion of patients using each antibiotic during pre-shortage and post-shortage periods | Descriptive statistics, Mann-Whitney U-test, chi-square test. Fisher's exact test | Cefazolin use decreased by 23%, ampicillin/sulbactam use increased by 21%, benzylpenicillin use increased by 19% |
| 55 | Proportion of patients using norepinephrine in shortage hospitals defined as any quarterly interval in 2011 during which the hospital rate of norepinephrine use decreased by more than 20%from baseline | Chi-square test, Wilcoxon rank test, and Kruskal-Wallis | Norepinephrine use decreased by 21.3% in the second quarter of 2011, and phenylphrine use increased by 18.2% in shortage hospitals |
| **Grey Literature Reports** | | | |
| 56 | Percent change in the number of active public drug plan beneficiaries during shortages, the proportion of shortages with no decline, 0-20% decline and more than 20% decline | NA | 23% of shortages had less than 20% decline in beneficiaries, 40% of shortages were associated with decline of greater than 20% |
| 57 | Average annual change in volume of oncology sterile injectables in pre-shortage period of 2006-2008 and in post-shortage period of 2008-2011 for 44 drugs that faced a shortage and 28 that did not | NA | Shortage oncology injectables had a 2.0% decline post-shortage and non-shortage oncology injectables had a 10.5% increase post-shortage |
| 58 | Changes in volume, prices, and patient counts around the start month of the shortage, and over 12 months in either direction with a washout period excluding the shortage start month | Descriptive statistics | On average, volume declined by 27.8 percent, price increased 16.6 percent, and unique patients declined by 10.8 percent across shortage drugs |
| 59 | Changes in the number of patients using shortage drugs and alternative drugs during pre-shortage and post-shortage periods | T-test | During their respective shortages asparaginase use decreased by 11 patients, nelarabine use increased by 30 patients and heparin use decreased by 934. |
| 60 | Changes in volume, prices, manufacturer count, and Herfindahl-Hirschman index for US shortages for the start month and 12 months in either direction, and changes across Canada, France, Germany, Italy, Japan, UK, US and Australia for shortages that aligned with US shortages. | Descriptive statistics and logistic regression analysis | On average, volume for shortage drug forms decreased with magnitudes ranging from 4.9 to 8.7 percent, compared with less than 2% increases for non-shortage drugs for the US. For other countries, we found an average 8.4-percent decline in volume compared with a 1.0-percent increase for non-shortage drugs. |
